# An Autonomous Chemical Robot Discovers the Rules of Inorganic Coordination Chemistry without Prior Knowledge

**DOI:** 10.1002/anie.202000329

**Published:** 2020-05-18

**Authors:** Luzian Porwol, Daniel J. Kowalski, Alon Henson, De‐Liang Long, Nicola L. Bell, Leroy Cronin

**Affiliations:** ^1^ School of Chemistry The University of Glasgow Glasgow G12 8QQ UK

**Keywords:** algorithms, artificial intelligence, autonomous discovery robot, supramolecular chemistry

## Abstract

We present a chemical discovery robot for the efficient and reliable discovery of supramolecular architectures through the exploration of a huge reaction space exceeding ten billion combinations. The system was designed to search for areas of reactivity found through autonomous selection of the reagent types, amounts, and reaction conditions aiming for combinations that are reactive. The process consists of two parts where reagents are mixed together, choosing from one type of aldehyde, one amine and one azide (from a possible family of two amines, two aldehydes and four azides) with different volumes, ratios, reaction times, and temperatures, whereby the reagents are passed through a copper coil reactor. Next, either cobalt or iron is added, again from a large number of possible quantities. The reactivity was determined by evaluating differences in pH, UV‐Vis, and mass spectra before and after the search was started. The algorithm was focused on the exploration of interesting regions, as defined by the outputs from the sensors, and this led to the discovery of a range of 1‐benzyl‐(1,2,3‐triazol‐4‐yl)‐*N*‐alkyl‐(2‐pyridinemethanimine) ligands and new complexes: [Fe(L^1^)_2_](ClO_4_)_2_ (**1**); [Fe(L^2^)_2_](ClO_4_)_2_ (**2**); [Co_2_(L^3^)_2_](ClO_4_)_4_ (**3**); [Fe_2_(L^3^)_2_](ClO_4_)_4_ (**4**), which were crystallised and their structure confirmed by single‐crystal X‐ray diffraction determination, as well as a range of new supramolecular clusters discovered in solution using high‐resolution mass spectrometry.

## Graphical abstract

**A chemical robot** was designed, assembled and utilised for the discovery of supramolecular architectures through the exploration of a chemical space exceeding 10^9^ possible reactions. By searching for reactivity differences, a range of new 1‐benzyl‐(1,2,3‐triazol‐4‐yl)‐*N*‐alkyl‐(2‐pyridinemethanimine) ligands were found and four new complexes of Fe and Co were discovered, autonomously discovering the rules of self‐assembly for these systems.

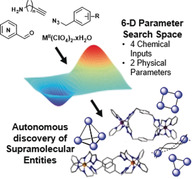

Exploring supramolecular chemical space for new assemblies is difficult not only because it is vast and sparse, but because we are looking for novel outcomes not easily predictable from a given set of input reagents and conditions. In addition, most reactions are done with specific possible outcomes in mind or rely on established heuristics. This means that the discovery of new and unpredicted coordination complexes is challenging and often occurs serendipitously.^1^ One approach to overcome this problem is by expanding the chemical parameter space to include, but to not be limited to, the theoretical optimal synthesis conditions of a known set of chemicals and look for unpredicted outcomes.^2^ However, expanding the scope of possibilities can result in an exponential increase in the number of possible experiments, along with a high cost in terms of time and resources. It is possible to use automation and suitable exploration algorithms to overcome these shortcomings.^3^, ^4^, ^5^, ^6^ A key question in terms of chemistry is how to efficiently synthesise a wide range of ligands and complexes,^7^ and then how to explore^8^ and understand the self‐assembly process.^9^, ^10^ An increase in the complexity of coordination compounds can be observed when two or more donor groups per ligand are spatially separated far enough from each other to avoid coordination with the same metal ion.^11^ Therefore, the ligand has to be structurally rigid so that the different binding sites prefer to coordinate in a bridging mode.^12^ It is also important that the ligand is flexible so that the coordination sites of the ligand and metal can adapt to each other.^13^, ^14^ Using these considerations it is possible to select a chemical space with many potential complexes to discover. Herein we present a fully automated supramolecular discovery platform that can both discover the ligand (by synthesis) and perform the complexation reaction an order of magnitude faster (2 h vs. 20 h) than other approaches due to the completely autonomous, closed‐loop nature of the system (see Figure 1).^15^

**Figure 1:**
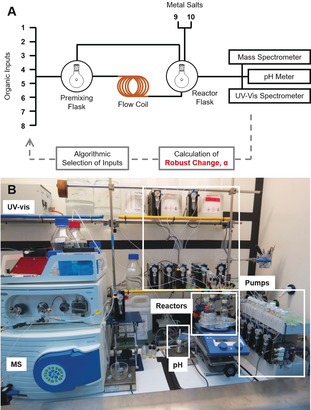
A) Connection diagram with the prominent connections of the system highlighted. B) Photograph of the system with major features annotated.

Not only are the reaction operations automated,^16^ but so is the process of discovery.^17^ To have flexibility to explore supramolecular self‐assembly, we aimed at the synthesis of a modified pyridyl‐triazole which can be broken into four parts giving a large library of potentially synthesisable ligands (see Scheme 1).^19^ However, the current syntheses of such frameworks can be very time consuming taking more than a day.^20^ The system we designed is capable of exploring a huge range of possible self‐assemblies constructed from three ligand classes (totaling 56 ligands) and two different metals. This exploration is algorithm‐driven and closed‐loop, running a sequence of experiments under an autonomous regime, which increases the chances (in a reasonable time scale) of the discovery of new compounds.

**Scheme 1:**
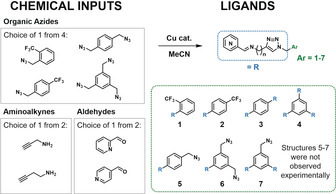
Small organic molecules used as chemical inputs for the synthesis of ligands, via tandem CuAAC and imine formation reactions, prior to complexation.

The autonomous decision making^21^ in this system focuses on the exploration of the most interesting regions of the chemical space by using live experimental data of reaction mixtures. For a robust definition of interest, we use a measure of the change that has occurred. The chemical space is defined by a selection of the starting materials consisting of the potential ligand building blocks and transition metal ions, and by three different reaction parameters: i) reagent volumes; ii) reaction temperature; and iii) reaction duration. To exemplify this idea, we had to establish some synthetic constraints, so we aimed to explore a potential ligand system with a new coordination motif (see Scheme 1). The synthesis was carried out as a three‐component reaction by combining one pyridinecarboxaldehyde (from two possibilities), one aminoalkyne (from a set of two), and one azide (from a set of four), each with a selected volume, for a chosen duration and temperature. The full set of choices results in 394 million possible reactions, demonstrating that even these limited inputs define a vast number of potential experiments.

The reactor for the synthesis step consists of a catalytically active 10 mL capacity copper coil,^22^, ^23^ which allows for the synthesis of the bespoke ligand system within a short duration of under 2 h (ligand dependent). This activated reactor promotes the coordination of the in situ formed ligand with leached Cu. The ligand formation happens due to full or partial imine condensation and/or copper‐catalysed alkyne–azide cycloaddition (CuAAC),^24^ depending on the reaction temperature and reaction duration. The experimental space is seven dimensional as it is defined by the reagents chosen (one aldehyde, one amine, one azide, and one metal), the reagent volumes used (from 0.5 mL to 5 mL each), the reaction duration (from 5 to 120 min), and temperature (from 30 to 80 °C). The different possible combinations of ligand precursors can make 56 potential ligands (see Scheme 1). The metal‐exchange reaction occurs once the ligand mixture collected from the catalytically active reactor (without purification) undergoes a second reaction step, consisting of complexation with a chosen volume of one of two metal salt solutions—[Fe^II^(ClO_4_)_2_] or [Co^II^(ClO_4_)_2_]. Including the second coordination step, the number of possible experiments in the chemical system increases to 4×10^14^. The cleaning, reaction activation, and decision‐making operations are all fully automated, allowing the continuous operation of the system without human involvement. The system is controlled using bespoke code written in Python such that the chemical robot can perform all the liquid‐handling operations, including cleaning procedures between one experiment and the next, as well as control of analytical instruments and data analysis. The design and the connectivity of the system is shown in Figure 1.

The routing of chemicals and solutions through the system is conducted in a non‐deterministic fashion (see Figure 2).^25^ This means that the paths through which the solutions are transported in the system are not predetermined. For each processing step the system only knows which solution is needed and where but not how to perform the operation. Instead the system decides, using graph operations on the network connection graph that describes the system. It chooses which route stochastically (see Supporting Information) to utilise, and optimises its own route for each single material transfer operation. The main benefits of such a system are increased flexibility, reduced complexity, and high scalability. Such systems can even work around faults arising in real time.

**Figure 2:**
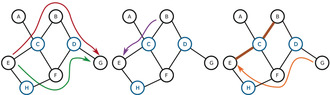
A diagram depicting the connections in our system as a network graph. Nodes depict either locations (in black) or valves (in blue), and the edges signify the physical connections between the nodes. Left: Two equivalent paths, in red and green, to move from node E to node G, the system will randomly choose one of them. Middle: The shortest path to move from node B to node E. Right: If the connections between nodes E and C, and C and B are not clean (shown in brown), for example, in response to the movement of material from B to E (see middle panel), the system chooses a new route. This is shown in orange, for movement from node G to node E, avoiding the unclean route from B to E.

In order to investigate how best to make discoveries in an experimental system, we designed both the chemical space and the exploration algorithm to not rely on *any* knowledge beyond the initial choice of the ligand reaction envelope.^15^ These areas may or may not contain discoveries, but the algorithm was designed to focus exploration to these regions preferentially. The algorithm does not build a model of the space during its exploration through it. In this way the system is designed to search in a stochastic manner, without biases or heuristics, yet to find areas of interest. Also, rather than optimise^26^ the reactivity, the system is coded to find as many interesting points in a given space. To be able to evaluate each experiment as a data point in the chemical space, the algorithm uses a live data stream from three sensors (UV‐Vis, mass spectrometry, and pH) to construct a simple and robust measure of the change occurring over both ligand synthesis step, and the metal ion coordination step (see Supporting Information). Therefore, the autonomous exploration of the chemical space is driven by the degree of change from the starting materials to the ligand and between the ligand and the coordination complex. This is characterised by an exploration factor, α, which is inversely proportional to the amount of change calculated, which, in turn, is related to reactivity or the degree to which a reaction has occurred. For a low α case the exploration algorithm will result in the next experiment being close in parameter space to the previous one, as this indicates a region of high reactivity. Conversely, if the change was small (i.e. high α and low reactivity) the system will search further away, in order to actively seek areas with more change. It is possible for the system to get stuck, so if too many experiments are performed in a small region, the system is programmed to jump to an unexplored part of the experimental space far away. In this way the algorithm is designed to perform a minimum number of experiments in any given area of interest to maximise the space explored.

An explanation of the principles of operation of the exploration algorithm is shown in Figure 3, which shows an illustration of the algorithm exploring a 2D space with color representing the low change areas (blue) to high (red). The first experiment in the sequence is a random point yielding a moderate value of difference change leading to a corresponding value of α_1_, where α is the radius of a circle around the point. The next experiments selected by the algorithm are points in the parameter space on this circle line, with the choice of point two (see Figure 3, panel B) from these being stochastic, as the algorithm knows nothing about the space. The new experiment will become the center of the new circle having a well‐defined radius of α_2_, and so on (see Figure 3, panels C and D). It is important to note that the system is programmed to have no more than three consecutive experiments in the same region of space, in order to avoid exploring the same local area in a loop.

**Figure 3:**
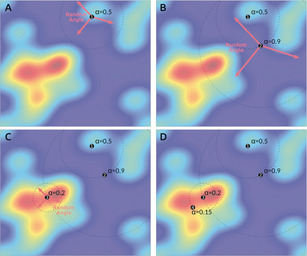
Schematic of the exploration algorithm operating in 2D space. The first point is chosen at random (A). The measured exploration factor, α for Point 1 determines the radius away at which Point 2 is placed (panel B). The larger the value of α, the larger the radius to the next point. This stepwise exploration is continued in panels C and D. Note that, over time, the exploration is concentrated to areas of higher chemical interest (shown as red).

The calculation of α is based on the ratio of differences between the analytical measurements of the ligand with its starting materials; and between the ligand and its resulting complex. Thus, this value constrains the exploration range for the next randomly chosen experiment. The calculation of the differences for each analytical measurement is detailed in the Supporting Information. In general, the differences are all based on the comparison of the two data sets—the ligand mixture and starting materials; or complex combination and ligand mixture. Specifically, MS changes are calculated using the most intense and highest *m*/*z* peaks found in each of the two spectra compared; UV‐Vis changes are measured as the difference of the two spectra compared after normalisation by calculating the root mean square value of the peak area difference; pH changes are evaluated using the pH of the starting material or of the ligand as a reference value (see Supporting Information).

Using this system we have been able to autonomously synthesise a range of 1‐benzyl‐(1,2,3‐triazol‐4‐yl)‐*N*‐alkyl‐(2‐pyridinemethanimine) ligands which were applied in the discovery of new complexes: [Fe(L^1^)_2_](ClO_4_)_2_ (**1**); [Fe(L^2^)_2_](ClO_4_)_2_ (**2**); [Co_2_(L^3^)_2_](ClO_4_)_4_ (**3**); [Fe_2_(L^3^)_2_](ClO_4_)_4_ (**4**). Exact details for the conditions used in the syntheses of any isolated compounds can be found in the Supporting Information. Complex **1** is formed through tridentate coordination of each ligand to Fe^II^, with one N‐donor atom from each of the pyridine, imine, and triazole groups. Despite L^1^ being more flexible than L^2^ (due to the additional methylene group), the favored coordination mode is still tridentate with all nitrogen containing moieties bound. In the UV‐Vis spectrum of the complex mixture (Figure S11), three new absorbance bands are detected at 356, 476, and 544 nm. This evidence lets us conclude that the intermediate [Cu(L^1^)_2_]^2+^ formed in the flow reactor undergoes a metal‐exchange process in the presence of the Fe^II^ salt to give [Fe(L^1^)_2_](ClO_4_)_2_. Also, the pH values and the ESI‐MS spectra of the two analysed mixtures are in accordance with this observation. In fact, a significant change in the acidity of the new complex from 6.29 to 4.34 is detected. The ESI‐MS shows the presence of peaks with *m*/*z* of 390.23 in the ligand mixture and *m*/*z* of 387.25 in the complex mixture. These could be assigned to [Cu(L^1^)_2_]^2+^ and **1** respectively.

Using this same methodology [Fe(L^2^)_2_](ClO_4_)_2_ (**2**) was discovered from an alternate set of input reagents yielding the general ligand framework 1‐benzyl‐(1,2,3‐triazol‐4‐yl)‐*N*‐alkyl‐(2‐pyridinemethanimine). Solid‐state structures of these complexes are shown in Figure 4. Further exploration of the space led to the isolation of helicates [Co_2_(L^3^)_2_](ClO_4_)_4_ (**3**) and [Fe_2_(L^3^)_2_](ClO_4_)_4_ (**4**) forming an M_2_L_2_ complex using a ligand based on a structure 3 type motif.

**Figure 4:**
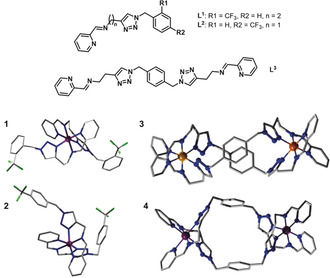
Crystal structures of the isolated compounds. Skeletal structures of ligands L^1–3^; **1**: [Fe(L^1^)_2_](ClO_4_)_2_; **2**: [Fe(L^2^)_2_](ClO_4_)_2_; **3**: [Co_2_(L^3^)_2_](ClO_4_)_4_; **4**: [Fe_2_(L^3^)_2_](ClO_4_)_4_.

The autonomous analytics run on these reaction mixtures show that this way of conducting experiments can generate a huge variety of different complexes in solution, not only because of the range of potential coordination modes of the ligands, but especially because more than one ligand can be present in the reaction solution and reactions may be performed with non‐standard reagent stoichiometries. This is demonstrated by the fact that we could isolate compound **4** through crystallisation from the reaction solution but the ESI‐MS measurements of the reaction mixture reveals more species to be present in solution (see Figure 5).

**Figure 5:**
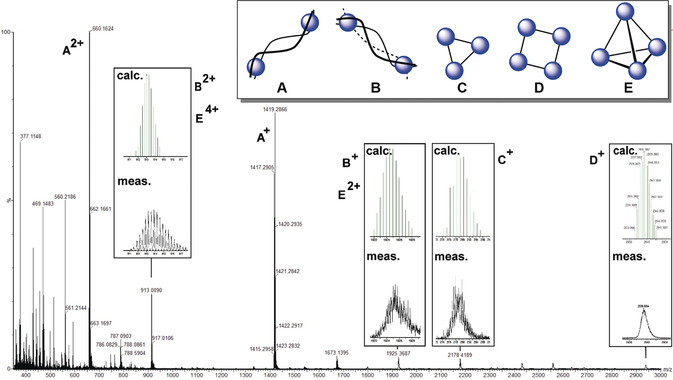
ESI‐MS detection of different possible structures based on the helicate complex **4** (noted here as architecture **A**). Architectures **B** and **E** have the general formula M_2_L_3_ and would be required to adopt κ^2^‐coordination through the pyridyl and imine groups. Architectures **C** and **D** have the general formula M_2_L_2_, as does **A**, and would adopt the same κ^3^‐coordination motif as the helicates shown in Figure 4. See Table 1 for the calculated and measured peak data.

All the assumed adducts are also summarised in Figure 5 and one set of signals for the compound indicated as **A** are consistent with the assumption that the tridentate motif coordinates two times per metal center to form the isolated helicate **4**. Changing the coordination mode can allow the formation of a triple‐stranded helicate (**B**) or a cage‐like arrangement (**E**). However, other peaks have *m*/*z* values that can be associated with the mass of specific cages, such as circular triangular (**C**) or square (**D**) arrangements (see Table 1). Arrangements like **C** and **D** may be possible because of the flexibility of ligand L^3^. However, to obtain those structures the conformation of the ligand (especially for the tridentate motif) must change to a bidentate motif. This makes it possible that three bidentate motifs are coordinated to one metal center to form the proposed structures illustrated in Figure 5. These results also suggest that the bipodal ligands can coordinate with their different coordination moieties in bidentate or tridentate fashion with the imine triazole unit excluding or including the pyridine one. The competition of two different ligand conformations might be a reasonable explanation for these observations. By MS, we can clearly distinguish masses that can be associated to three different proposed structures in solutions and assumed to be formed from the same ligand and Fe^II^ under one reaction condition.

**Table 1:** Calculated and measured mass spectra peaks.

Peak	Proposed cation	*m*/*z*		
		charge	calculated	measured
**A**	[Fe_2_(L^3^)_2_](ClO_4_)_4_	1+	1420.22	1420.29
		2+	660.14	660.16
		3+	407.45	407.46
		4+	280.60	280.11
**B**	[Fe_2_(L^3^)_3_](ClO_4_)_4_	1+	1924.47	1924.73
		2+	912.77	912.29
		3+	575.53	575.19
		4+	407.45	407.16
**C**	[Fe_3_(L^3^)_3_](ClO_4_)_6_	+	2178.30	2177.85
		2+	1040.18	–
		3+	660.14	660.48
		4+	469.75	469.14
		5+	357.11	–
		6+	280.16	280.11
**D**	[Fe_4_(L^3^)_4_](ClO_4_)_8_	1+	2938.39	2938.09
		2+	1420.22	1420.29
		3+	912.77	912.29
		4+	660.14	660.48
		5+	508.93	–
		6+	407.45	407.16
		7+	335.39	–
		8+	280.16	280.11
**E**	[Fe_4_(L^3^)_6_(ClO_4_)_8_	1+	3946.89	–
		2+	1924.47	1924.47
		3+	1248.6	–
		4+	912.77	912.29
		5+	710.63	–
		6+	574.75	575.86
		7+	478.4	–
		8+	406.25	407.29

In summary we have achieved the exploration of a vast range of potential experiments without any heuristics or other chemical knowledge. Using this approach, the system actively searches through the available space based on the information obtained about the system as it is running. The ligand system targeted has not been previously reported in the literature, although it is simple and can lead to many outcomes. Furthermore, to the best of our knowledge, no coordination architectures are known to be self‐assembled through the presented coordination moiety. Our studies show the complexity of this ligand system in the outcome of several Co^II^‐ and Fe^II^‐ coordination architectures. The screening of a huge chemical space with the described ligand system resulted in four new coordination structures, which could be isolated, and their molecular structures could be ascertained by X‐ray diffraction.

## Experimental Section

A volume explaining the platform, software, search data, analytical data and crystallographic data is available. CCDC 1529980, 1529981, 1529982 and 1529983 (**1**–**4**) contain the supplementary crystallographic data for this paper. These data can be obtained free of charge from The Cambridge Crystallographic Data Centre. Code is also available from the Croninlab git pages: https://github.com/croningp/InorganicFinder.

## Conflict of interest

The work in this publication has been filed as a patent by the University of Glasgow.

## Supporting information

As a service to our authors and readers, this journal provides supporting information supplied by the authors. Such materials are peer reviewed and may be re‐organized for online delivery, but are not copy‐edited or typeset. Technical support issues arising from supporting information (other than missing files) should be addressed to the authors.

Supplementary
