# Supplementary material for: An Autonomous Chemical Robot Discovers the Rules of Inorganic Coordination Chemistry without Prior Knowledge
Source: Angew Chem Int Ed Engl. 2020 May 18;59(28):11256–61. doi: 10.1002/anie.202000329 (PMC7384156; doi:10.1002/anie.202000329)
Supplement: Supplementary file 1 — Supplementary [file ANIE-59-11256-s001.pdf]

Angewandte Chemie
Eine Zeitschrift der Gesellschaft Deutscher Chemiker

Supporting Information

An Autonomous Chemical Robot Discovers the Rules of Inorganic Coordination Chemistry without Prior Knowledge

*Luzian Porwol, Daniel J. Kowalski, Alon Henson, De-Liang Long, Nicola L. Bell, and Leroy Cronin**

anie_202000329_sm_miscellaneous_information.pdf

SUPPLEMENTARY INFORMATION

Table of contents

1 EXPLORATION ALGORITHM AND CHEMICAL SPACE 3
1.1 ROBOT PROTOCOL ONTOLOGY 3
1.2 OPERATION SUMMARY 4
1.3 DESCRIPTION OF CHEMICAL SPACE 5
1.4 EXPLORATION ALGORITHM 5
1.5 CALCULATIONS FOR CHEMICAL SPACE 8
1.5.1 Calculation of the Size of the Chemical Space 8
1.5.2 Calculation of the Total Reaction Time 8
1.5.3 Calculation and Meaning of α 9
1.5.4 Calculation of Total Changes (Δ) 9
1.5.5 Calculation of Ligand reaction changes (Δ1) 10
1.5.6 Calculation of Complexation reaction changes (Δ2) 11
1.6 EXAMPLES OF CHEMICAL SPACE FOR DISCOVERIES 13
2 INSTRUMENTATIONS AND MATERIALS 14
2.1 MATERIALS 14
2.2 OFFLINE ANALYTICS 14
2.3 IN-LINE ANALYTICS 17
3 CHEMICAL ROBOT HARDWARE 18
3.1 HARDWARE SET-UP 18
3.1.1 Robot Operations 20

3.1.2 Temperature, Cross-Contamination and Reactor-Activation Control 23
3.1.3 Reliable automated acquisition of UV-vis data 24
3.1.4 Automated acquisition of pH-values 26
3.1.5 Example measurements for a single experiment 26
3.2 Software 27
3.2.1 Connections and Communication 27
3.2.2 Non-deterministic Routing 27
4 Syntheses 30
4.1 Azide Syntheses 30
4.2 Ligand Variety 32
4.3 Selected Ligand Syntheses 34
4.4 Complexes Discovered autonomously 43
4.4.1 Isolation of $[Cu(2\text{-pyridinecarboxyaldehyde})_2]_n$ 44
4.4.2 Isolation of $[Fe(L^1)_2](ClO4)_2$ (Complex 1) 45
4.4.3 Isolation of $[Fe(L^2)_2](ClO_4)_2$ (Complex 2) 46
4.4.4 Isolation of $[Co_2(L^3)_2](ClO_4)_4$ (Complex 3) 47
4.4.5 Isolation of $[Fe_2(L^3)_2](ClO_4)_4$ (Complex 4) 48
4.5 Observed Complexes 49
4.5.1 Observation of $[Fe(L^{13})_2](ClO4)_2$ (Complex 5) 49
4.5.2 Observation of $[Co(L^1)_2](ClO_4)_2$ (Complex 6) 50
5 Crystallographic Details 52
5.1 $[Fe(L^1)_2](ClO_4)_2\cdot(C_6H_{14})_{0.5}$ (1) 52
5.2 $[Fe(L^2)_2](ClO_4)_2\cdot$2CH2Cl2 (2) 53
5.3 $[Co_2(L^3)_2](ClO_4)_4\cdot 10CH_3OH$ (3) 54
5.4 $[Fe_2(L^3)_2](ClO_4)_4\cdot(CH_2Cl_2)_2$ (4) 55
6 References 56

1 Exploration algorithm and chemical space

1.1 Robot Protocol Ontology

The operations carried out by the chemical robot can be conceptually summarized as illustrated in the flow diagram in **Figure S1**.

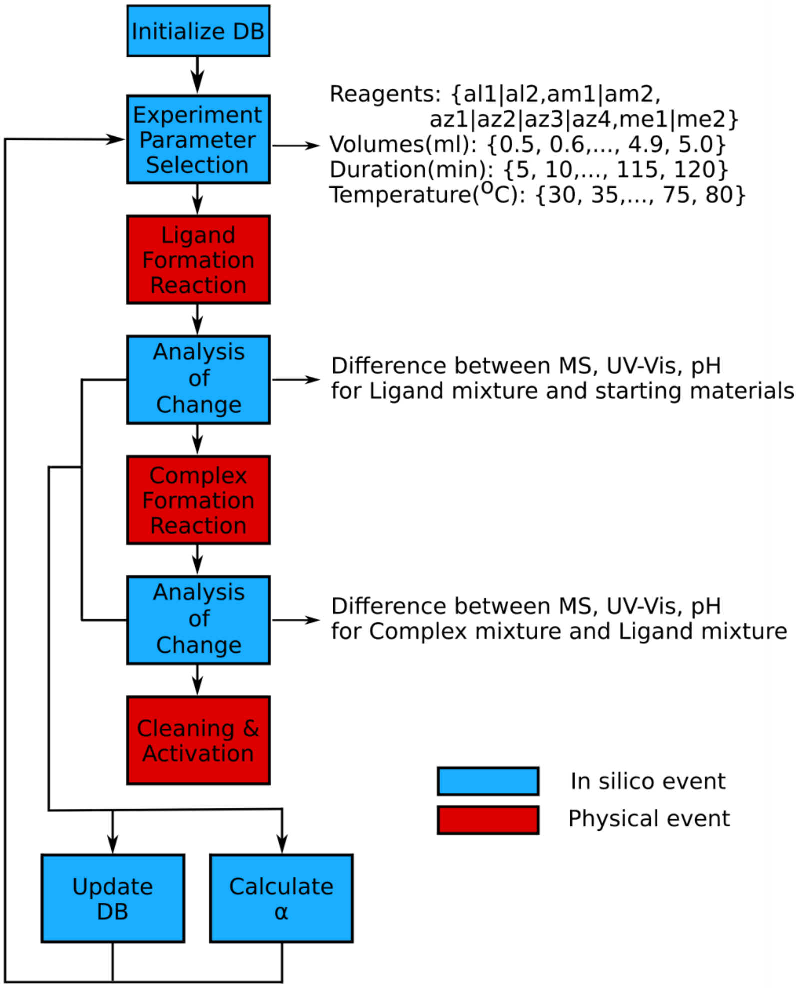

Figure S1 A flow diagram showing how the chemical robot autonomously looks for change and decides on the conditions of the next reaction to be performed – physical events are shown in blue and computational steps in red. A random starting point for the reaction parameters is selected and the ligand reaction is performed and then analyzed. This is then complexed with a metal salt and the mixture analysed once more. After the two steps are completed, the system starts the cleaning process and simultaneously calculates α by assessing the ratio of the differences between starting material/ligand spectra (Δ1); and ligand/complex data (Δ2). Once α is calculated, the new experimental parameters are set.

1.2 OPERATION SUMMARY

1 Load configuration file with information about the topology of the system (all connectivity of the pump inputs and outputs).

2 Load file with saved starting materials' MS, UV-Vis spectra and pH values.

3 Stochastic selection of the first experiment where all possibilities have the same probability to be chosen. Selection of reagent volumes, identity and reaction temperature and time is random.

4 Prepare the system for the reaction: heat copper coil reactor to the selected temperature, prime tubing with selected reagents, and collect the UV-vis absorbance spectra of the reagent solutions to use as reference.

5 Combine organic reagents in the first vial and move 10 mL this mixture to the coil (any excess is moved to waste).

6 After the selected reaction time, move10 mL of the reaction mixture from the coil to the second vial.

7 Move a sample of reaction mixture to a third vial, dilute with solvent and collect pH and UV.

- Dilute further if the spectra are saturated

8 Move another sample (via a distinct pathway) of reaction mixture to the MS.

9 Combine the remaining reaction mixture with the randomly selected metal salt (random volume up to 5 mL) in the vial containing the ligand reaction mixture.

10 Optionally, e-mail the operator that the reaction is finished and the reaction mixture can be collected if needed.

11 Repeat step 7 and 8 for this new reaction mixture.

12 Start the cleaning process and simultaneously calculate α (decision making algorithm in action).

13 Once α is calculated the system can autonomously select a new experiment.

- The value of α defines how far away from the current reaction condition the next experiment will be in parameter space. A small α indicates that the new conditions will be very similar to the previous, as this is a region of the chemical parameter space that shows a large change between ligand mixture and complex mixture data.
- The selection of the new experiment is again stochastic, with all possibilities having the same probability to be chosen, but the available chemical parameter space to choose from is smaller than in the initial experiment and is defined by α.

14 Repeat operations 4 to 13.

1.3 DESCRIPTION OF CHEMICAL SPACE

The exploration algorithm needs to be able to cope with the size and multidimentionality of the chemical space investigated. Most chemical search spaces are designed with a specific predicted outcome in mind. This means that the search process is either an optimization or screening of the possibilities. In order to promote discovery, we have selected a space that is not targeted towards any specific outcome. In addition to varying the reagent selection we also vary the parameters of volume, reaction duration and reaction temperature. This yields conditions that otherwise might be called non-ideal, yet in our system they allow for partial reactions to take place. This creates the potential for mixtures of several ligands to be formed for a given set of reaction conditions which are then made available for the subsequent complex formation. The resulting chemical space is extremely large, highly varied and unpredictable. The time it would take to conduct all the experiments, taking solely the reaction duration into account, is ~4.5 million years (see **Section 1.3.2**).

1.4 EXPLORATION ALGORITHM

The algorithm starts by randomly choosing an experiment in space with all the parameters equally probable. To evaluate the experimental result it collects the analytical data from multiple instruments for two reaction steps and then uses a simple comparison to quantify the amount of change that has occurred as described in detail in **Section 1.3.3**. The change calculation uses a relatively simple measure for robustness. More complicated measures might prove to be too reaction specific which would be detrimental for a space as varied as ours. The value of change is used as a measure of how interesting a point is in the space.

The basic principle of exploration is to utilize stochasticity and to modify the probability of choosing experimental points in space in order to increase the probability of performing experiments in regions of interest. A high value of change indicates that the local area is worth further exploration and the algorithm should choose a point that is in the vicinity of

the last one. With this goal in mind, the algorithm calculates a value that is the radius of a 6D sphere (as the chemical space is of 6 dimensions) and the next experimental point is chosen randomly from the envelope of this sphere. If the last point had a high value of change, the next experiment will be near to it in space, but if the value of change is low then the random choice would yield a point that is further from the last. For values of change that are not towards these extremes, the algorithm will choose a set of conditions that is correspondingly intermediate in distance.

A simplified example can be seen in **Figure S2** which shows a 2D chemical space in which a sequence of experiments is performed. The first point is entirely random and is therefore more likely to find an area that is of little interest. In 2D, the 6D stochastic sphere discussed above becomes a circle and the next experiment is randomly chosen from its circumference. For Point 1 the change is small and so α_1 is large, in order to move into a new area of the space. Point 2 is of more interest and its value of α is therefore smaller, so that the exploration is constrained to a closer region of the space. This trend of more interesting experiments leading to smaller α's and closer experiments continues through Point 3 followed by Point 4.

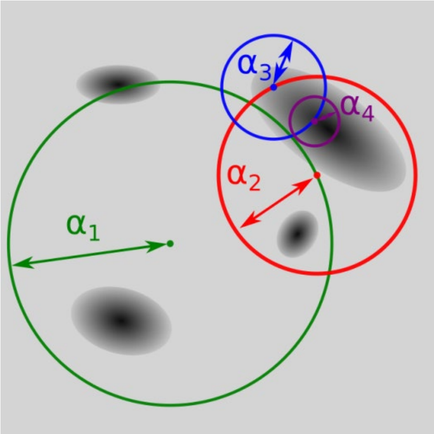

Figure S2 – A conceptual diagram of the exploration process in a 2D space. The dark regions are areas with large degrees of change with a gradient to a maximal values in their centres. The points in the middle of each circle indicate an experiment with a given set of conditions, and α is the distance at which the subsequent random selection is made. Shown is a sequence of 4 experiments and their resulting α.

Due to the practical limitations of performing experiments in the physical world with finite resources it is not reasonable to explore the space by using a deterministic method of exploration. Any method that builds a model of the space requires a certain level of knowledge to reach a useful level of understanding about the space. Given the size and unknown topology of our space, this is not feasible for the explorations detailed herein.

A major problem in any search algorithm is getting stuck in local maxima. Since the algorithm increases the probability of selecting subsequent experiments nearby once an interesting region is identified, there is a possibility of the exploration becoming 'trapped' to this region. In order to overcome this, the system must have an awareness of the relative locations in space of previous points. For that purpose, each experiment was saved to a database and every time the algorithm needs to make a decision about where to perform the next experiment it first loads the accumulated data. After the algorithm chooses a new point it will check if there are 3 or more other points in close vicinity. If that is the case the proposed point is rejected and the algorithm makes the selection again, see **Figure S3**. The choice now is made by evaluating the farthest distance that could be taken from the last performed point and that value is taken as the distance for the stochastic selection. This ensures that the next point is as far as possible from the previous one. It is important to note that there would usually be areas in the space that would not be accessible with this type of selection as most points are not equally distant from all edges, unless it is the exact middle point in 6 dimensions.

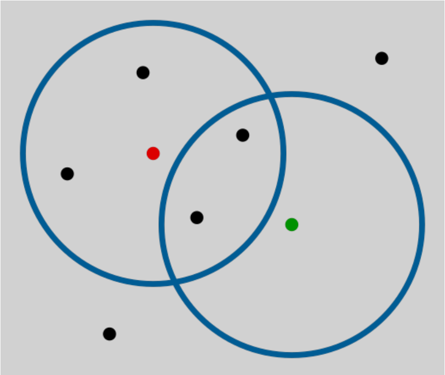

Figure S3 – Scheme of the exclusion area in a 2D space. The black dots mark the location of previous experiments, the red dot marks the next proposed experiment. Since this proposed point has in its vicinity (the blue circle) more than 3 previous experiments it is rejected. The green dot is an allowed point as it only has 2 prior experiments within its vicinity.

1.5 CALCULATIONS FOR CHEMICAL SPACE

1.5.1 Calculation of the Size of the Chemical Space

To calculate the size of the chemical space we need to take four factors into account. The reagent choice is the number of ways to choose a set of 4 chemicals out of the available number of options. In this case, there are 2 possible aldehydes, 2 possible amines, 4 possible azides and 2 possible metals. This results in 32 combinations each representing its own chemical space. Each chemical can have a volume between 0.5 mL to 5.0 mL with a resolution of 0.1 mL. This gives 46 different possible volumes per chemical. For the temperature the range is 30 – 80 ^{o}C in steps of 5 leading to 11 temperature options. Finally, reaction duration can have values from 5 min to 120 min with steps of 5 min, giving 24 possibilities. The combination of these parameters gives a six-dimensional space with one dimension for each of the four reagent volumes, reaction duration, and temperature. A small edge case occurs because the volume of the copper coil is limited to 10 mL so that random combinations of ligand precursors that yield above that volume still only transfer 10 mL to the reactor and the rest goes to waste. This is only a factor when the ratio between the three ligand precursors is identical and so we need to discount the possibilities of ligand starting materials having the same volume going up from 3.4 to 5.0 mL. Overall this is 17 possible experiments that would yield the same result. The full number of possibilities, over all possible 6D chemical spaces, taking all these factors into account is:

$$\left((46^4 \times 24 \times 11) \times 32\right) - 17 \cong 3.8 \times 10^{10} \qquad \text{(Eq. 1)}$$

1.5.2 Calculation of the Total Reaction Time

To indicate how large the total experimental space is we can calculate the time it would take to perform all the possible reactions. To do so we first need to calculate the number of possible experiments in five-dimensional space as the sixth dimension of reaction duration is the parameter we will be summing over.

$$(46^4 \times 11) \times 32 \cong 1.6 \times 10^{10} \quad \text{(Eq. 2)}$$

Now we can multiply this value with each one of the possible reaction durations and then sum them together:

$$(1.6 \cdot 10^{10} \times 5\,min) + (1.6 \cdot 10^{10} \times 10\,min) + \cdots = 2.3 \cdot 10^{10}\,min \cong 4.5 \cdot 10^6\,yrs \quad \text{(Eq. 3)}$$

1.5.3 Calculation and Meaning of α

The value of α gives a measure of the changes between the data collected from the analysis of starting materials and reaction products. The higher the value, the bigger the changes in reaction conditions required in the next experiment in order to direct the search to the most reactive region of the chemical space, which can be associated with the region having high probability of forming new complexes. It is mathematically inversely proportional to the sum of measured changes (see **Equation 4**).

$$\alpha = maximum\frac{threshold*1}{\Delta totalchanges} \quad \text{(Eq. 4)}$$

The maximum threshold is necessary since the total changes might vary immensely and the search space is finite in all dimensions therefore the algorithm must limit α to selecting points within the chemical space. This leads to the need for a normalization factor which takes into account the location of the most recent point. It is the largest distance possible in the space from that given point. This value changes for each point and is calculated every time **Equation 4** is performed.

1.5.4 Calculation of Total Changes (Δ)

The calculation of α is based on the difference among the solutions, and the calculation of these changes is based on the comparison of the MS, UV-Vis and pH data of the reaction mixtures resulting from the ligand, the complex and the initial starting materials selected. The difference measured from the changes in the MS, UV-Vis and pH values are first evaluated individually and then combined taking into account their informative value. Therefore, the three changes are combined in different weight percentages that have been empirically chosen to be 0.6 for MS, 0.3 for UV and 0.1 for pH.

$$\Delta totalchanges = (0.6 \times \Delta MS) + (0.3 \times \Delta UV) + (0.1 \times \Delta pH) \quad \text{(Eq. 5)}$$

In general, the changes are all based on the comparison of data – ligand mixture and starting materials, and complexation reaction and ligand mixture. The starting materials mixture is calculated virtually from saved measurement using a linear relation assumption and no interaction between chemicals. MS changes are calculated using the most intense and highest peaks found in each of the two spectra compared; UV-Vis changes are measured as the difference of the area of the two spectra compared; pH changes are evaluated using the pH of the starting materials or of the ligand as a reference value. **The total difference value** per each measurement (Δ) is intended as the ratio between **the difference calculated after the complex formation** (Δ2) and **the difference calculated after the ligand formation** (Δ1**,** see **Equations 6-8**).

$$\Delta MS = \frac{\Delta MS2}{\Delta MS1} \quad \text{(Eq. 6)}$$

$$\Delta UV = \frac{\Delta UV2}{\Delta UV1} \quad \text{(Eq. 7)}$$

$$\Delta pH = \frac{\Delta pH2}{\Delta pH1} \quad \text{(Eq. 8)}$$

1.5.5 Calculation of Ligand reaction changes (Δ1)

The calculation of the changes between the ligand and the starting material is detailed here:

- **delMS1 = ΔMS1** Select the biggest *m/z* with the highest intensity in the ligand mixture (peak L1) and in the starting material virtual mixture (peak M2).

  Then search for these peaks in the other spectra (M1 in ligand and L2 in starting material) and compare their intensity (margin value 3 = marg, see **Equations 9-14**).

$$ratio = \frac{intensityligand(L1)}{intensitymixture(M2)} \quad \text{(Eq. 9)}$$

$$M2_{max} = maxintensity \in range(M2 + marg)(M2 - marg) \quad \text{(Eq. 10)}$$

$$M1_{max} = maxintensityrange[(M1 - marg) \times ratio][(M1 + marg) \times ratio] \quad \text{(Eq. 11)}$$

$$L1_{max} = maxintensity \in therange(L1 - marg)(L1 + marg) \quad \text{(Eq. 12)}$$

$$L2_{max} = maxintensity \in range[(L2 - marg) \times ratio][(L2 + marg) \times ratio] \quad \text{(Eq. 13)}$$

$$\Delta MS1 = (M2_{max} - M1_{max}) + (L1_{max} - L2_{max}) \quad \text{(Eq. 14)}$$

- **delUV1 = ∆UV1** Is based on area differences calculated as root mean square error (RMSE). First the ligand spectrum is normalized for the maximum of the mixture of starting materials; then the ratio is calculated between normalized ligand and mixture; then the difference value is obtained as the RMSE of the previous values (**Equations 15-17**).

$$UV_{Ligand_{normalized}} = \frac{UV_{Ligand}}{UVmax_{Ligand}} \times UVmax_{mixture_{sm}} \quad \text{(Eq. 15)}$$

$$UV_{\Delta Ligand} = UV_{Ligand_{normalized}} - UV_{mixture_{sm}} \quad \text{(Eq. 16)}$$

$$\Delta UV1 = \sqrt{\frac{\sum_i^n (UV_{\Delta Ligand})^2}{n}} \quad \text{(Eq. 17)}$$

- **delpH1 = ∆pH1**

$$\Delta pH1 = \frac{pH_{Ligand} - pH_{mixture_{sm}}}{pH_{mixture_{sm}}} \quad \text{(Eq. 18)}$$

1.5.6 Calculation of Complexation reaction changes (Δ2)

The calculation of the changes between the complex and the ligand is detailed here:

- **delMS2 = ∆MS2** Select the biggest *m/z* with the highest intensity in the complex mixture (peak C1) and in the ligand mixture (peak L2). Then search for these peaks in area differences as root mean square error (RMSE). First the complex spectrum normalized at the maximum of the ligand one; then the ratio is calculated between the normalized complex and the ligand; then the difference value is obtained as the square RMSE of the previous values (see **Equations 19-21**).

$$UV_{Complex_{normalized}} = \frac{UV_{Complex}}{UVmax_{Complex}} \times UVmax_{Ligand} \quad \text{(Eq. 19)}$$

$$UV_{\Delta Complex} = UV_{complex_{normalized}} - UV_{ligand} \quad \text{(Eq. 20)}$$

$$\Delta UV1 = \sqrt{\frac{\sum_i^n \left(UV_{\Delta Complex}\right)^2}{n}} \quad \text{(Eq. 21)}$$

- **delpH2 = ∆pH2**

$$\Delta pH2 = \frac{pH_{Complex} - pH_{Ligand}}{pH_{Ligand}} \quad \text{(Eq. 22)}$$

1.6 Examples of Chemical Space for Discoveries

To show how distant the discoveries can be from the theoretically ideal input space we plot four compounds in a 3 dimensional depiction of the possible volume space in **Figure S4**. Although there are four different possible volumes of starting materials to consider we can treat the volume of Azide as a reference that does not need to be displayed. The volume of the Azide determines the ratio of the other starting materials as it comes in mono-, di-, and tri- substituted types and so the rest of the starting materials need to scale according to the type and volume of the Azide. For subplots C and D the possible volumes are larger by a factor of two since the Azide type is a di-azide. The Euclidean distances between the real and ideal points A to D are, respectively, 2.9, 0.7, 9.8 and 4.6 mL. For comparison the maximal volume difference in the system for a single starting material is 4.5 mL.

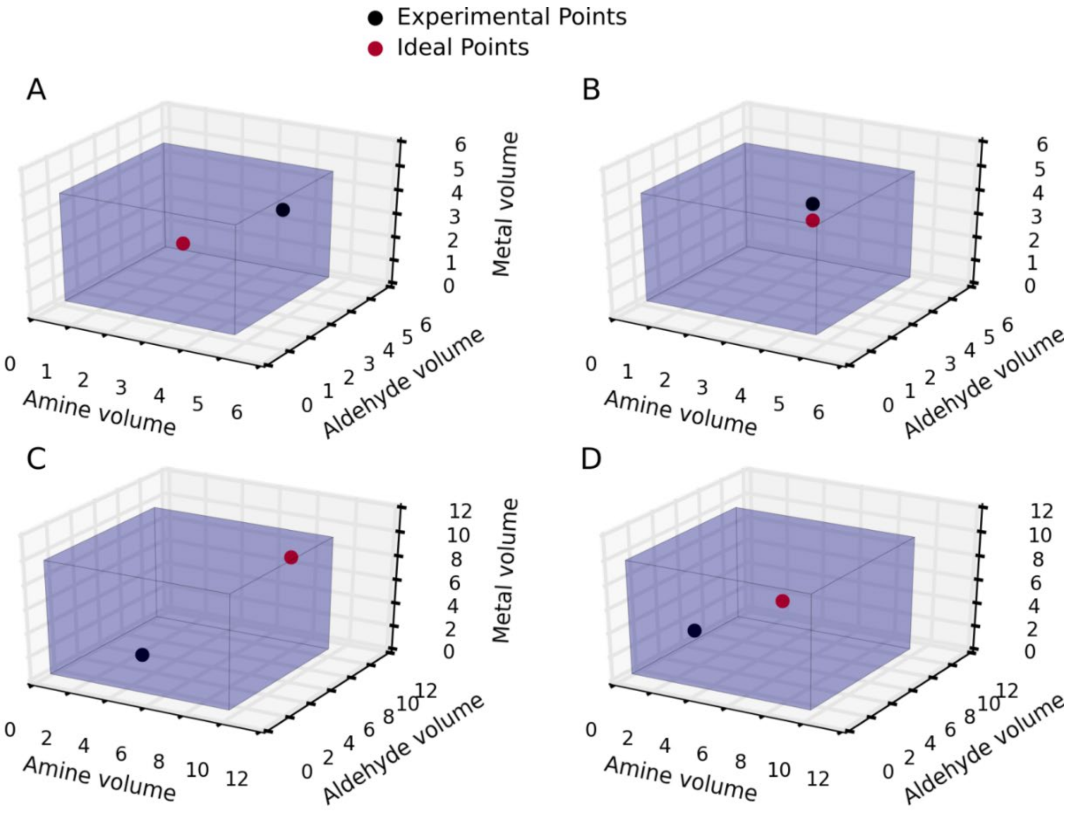

Figure S4 – Subplots showing 4 different discoveries in the volume space. Subplots A-D correspond to compounds 1-4 respectively. The blue cube marks the possible space for the three starting materials Amine, Aldehyde and Metal. The red points are the ideal mixtures based on the type and volume of Azide. The black points are the real volumes of the experimental discoveries.

2 INSTRUMENTATIONS AND MATERIALS

2.1 MATERIALS

All chemicals were supplied by *Fisher Chemicals*, *Sigma Aldrich* and *Lancaster Chemicals Ltd*. and were used without further purification. Solutions were freshly prepared before each experiment. Solvents for synthesis (AR grade) were supplied by *Fisher Chemicals* and *Riedel-de Haen*. Deuterated solvents were obtained from *Goss Scientific Instruments Ltd.* and *Cambridge Isotope Laboratories Inc*. PTFE tubing with different internal diameters, PEEK connectors and manifolds were supplied by *Kinesis* (Kinesis Ltd.). Copper tubing was supplied by RESTEK.

2.2 OFFLINE ANALYTICS

NMR spectroscopy: NMR data was recorded on a Bruker Advanced 400 MHz or a Bruker Advanced 600 MHz spectrometer. ^{1}H-NMR at 400/600 MHz and ^{13}C-NMR at 100 MHz, in deuterated solvent, at T = 298 K, using TMS as the scale reference. Chemical shifts are reported using the δ-scale, referenced to the residual solvent protons in the deuterated solvent for ^{1}H and ^{13}C-NMR (*i.e.* ^{1}H: δ (CD_3CN) = 1.96 ppm; ^{13}C: δ (CD_3CN) = 118.00 ppm; ^{1}H: δ (DMSO) = 2.50 ppm; ^{13}C: δ (DMSO) = 39.52 ppm; ^{1}H: δ ($CDCl_3$) = 7.26 ppm; ^{13}C: δ ($CDCl_3$) = 77.00). All chemical shifts are given in ppm and all coupling constants (*J*) are given in Hz as absolute values. Characterization of spin multiplicities: s = singlet, d = doublet, t = triplet, q = quartet, m = multiplet, dd = doublet of doublets etc.

Electrospray Ionization-Mass Spectrometry (ESI-MS): Measurements were carried out at 180 °C in water using a Bruker MaXis Impact instrument. The calibration solution used was Agilent ESI-L low concentration tuning mix solution, Part No. G1969-85000, enabling calibration between approximately 50 *m/z* and 2000 *m/z*. Samples were dissolved in water and introduced into the MS at a dry gas temperature of 180 °C. The

ion polarity for all MS scans recorded was negative, with the voltage of the capillary tip set at 4500 V, end plate offset at −500 V, funnel 1 RF at 400 Vpp and funnel 2 RF at 400 Vpp, hexapole RF at 200 Vpp, ion energy 5.0 eV, collision energy at 15 eV, collision cell RF at 2100 Vpp, transfer time at 120.0 μs, and the pre-pulse storage time at 20.0 μs. Mass spectrometry data was analysed using Data Analysis 4.0 software supplied by Bruker Daltonics.

Crystallography: Suitable single crystals were selected and mounted onto a rubber loop using Fomblin oil. X-ray diffraction intensity data was collected on a Bruker Apex 2 CCD diffractometer (λ (MoKα) = 0.71073 Å) equipped with a microfocus x-ray source (50 kV, 1.00 A). Data collection and reduction were performed using the Apex2 software package and structure solution, and refinement were carried out using SHELXS-97[1] and SHELXL-97[2] using WinGX suite[3] or OLEX2[4] Corrections for incident and diffracted beam absorption effects were applied using empirical absorption correction.[5] Most of the non-hydrogen atoms (including those disordered) were anisotropically refined. The SQUEEZE Function of Platon[6] was used on the structures for **3** & **4** to remove 10 MeOH and 1.2 DCM molecules respectively. CCDC 1529980−1529983 contain the supplementary crystallographic data for compounds **1-4**. These data can be obtained free of charge via www.ccdc.cam.ac.uk/data_request/cif, or by emailing data_request@ccdc.cam.ac.uk, or by contacting The Cambridge Crystallographic Data Centre, 12 Union Road, Cambridge CB2 1EZ, UK; fax: +44 1223 336033.

GC-MS measurements

Gas chromatography mass spectrometry (GC-MS) analysis was performed using an Agilent Technologies 7890A GC system equipped with Agilent Technologies 5975C inert XL MSD with Triple-Axis Detector. The column used was Agilent 19091N-102: 260 °C, 25 mm x 200 m μx 0.2 μm wide bore.

CNH Microanalysis: Carbon, nitrogen and hydrogen content (%) were determined by the microanalysis services within the School of Chemistry, University of Glasgow using an EA 1110 CHNS CE-440 Elemental Analyzer.

Computer controlled hot plate: IKA RET control. Integrated temperature control enables connection of a temperature probe, placed directly in the medium, to control its temperature with a high degree of precision. PT 100 temperature sensor was used. The stainless steel composite hot plate, reaching a temperature of 340 °C, enables rapid heating. RS 232 interface enable PC control of the magnetic stirrer, heating function and recording of all current parameters. A locking function prevents inadvertent changes of speed and temperature settings.

Syringe pumps: Liquid handling was performed using C3000 model, TriContinent™ pumps (Tricontinent Ltd, CA, USA) equipped with 5 mL syringes (TriContinent™) and 3-way solenoid valves (TriContinent™). Pump accuracy tests/results are reported in **Table S1** and were carried out with water.

Table S1 Tricontinent syringe pump accuracy test with average error percentage.

Set value (mL)	Measured value (mg)	Individual error (%)	Average ± error (%)
**0.01**	0.0059<br>0.0056<br>0.0063	41.0<br>44.0<br>37.0	**40.5 ± 3.5**
**0.05**	0.0423<br>0.0426<br>0.0422	15.4<br>14.8<br>15.6	**15.2 ± 0.4**
**0.1**	0.0913<br>0.0924<br>0.0922	8.7<br>7.6<br>7.8	**8.15 ± 0.55**
**0.25**	0.2417<br>0.2435<br>0.2434	3.32<br>2.6<br>2.64	**2.96 ± 0.36**
**0.50**	0.4938<br>0.4916<br>0.5081	1.24<br>1.68<br>1.62	**1.46 ± 0.22**
**1.00**	0.9927<br>0.9940	0.73<br>0.60	**0.665 ± 0.065**

2.3 IN-LINE ANALYTICS

Bench-top MS spectrometry: The spectra were recorded using a Microsaic systems 4000 MiD, spraychip® (electrospray ionization source). Masscape® software was used for control of sampling methods and manual data analysis. The specifications of this spectrometer are listed below:

Mass analyzer	ionchip® quadrupole mass spectrometer
Direct flow rate	0.2 μL min^{-1}– 2 μL min^{-1}
Split flow rate	up to 2.0 mL min^{-1}
Make-up flow	1 mL min^{-1}, 50 : 50 MeOH : H_2O
Attenuation	1000
Ionisation mode	positive
Tip voltage	850 V
Nebulizer (N_2) flow	2.5 L min^{-1}
Vacuum interface voltage	40 V
Tube lens voltage	10 V
Plate lens voltage	5 V
Ion guide voltage	1 V
Count time	0.20 ms
Mass range	*m/z* 50-800 with ionchip®150
Mass accuracy	+/- *m/z* 0.3 in full scan
Mass resolution	*m/z* 0.7 +/- 0.1 FWHM

UV-Vis spectroscopy: UV-vis spectra were acquired with a DH-2000 light source and a flow cell FIA-Z-SMA 905 (10 mm path length) from Ocean Optics, connected by fibre optics to an AvaSpec 2048 from Avantes. Spectra were collected every 1-2 seconds employing a customized program and processed employing an in-house developed program with Python and LabVIEW™.

pH meter: VWR universal pH and redox electrode supplied with a durable epoxy body shaft sealed gel-filled reference designs. This is also supplied with ceramic diaphragm and fixed cable. Diameter and length are 12 mm and 120 mm respectively.

3 CHEMICAL ROBOT HARDWARE

3.1 HARDWARE SET-UP

All liquid handling was undertaken by TriContinent™ pumps equipped with 5 mL syringes and 3-way solenoid valves. All syringe pumps were equipped with PTFE tubing for delivering the reagents into the reactors and moving the solutions in the system. All in line analytics were physically connected to a computer by a USB to a daisy chained serial connection. Schematics of the chemical robot are illustrated in **Figures S5** and **S6**, with specifications shown in **Table S2** and a photograph presented as **Figure S17**.

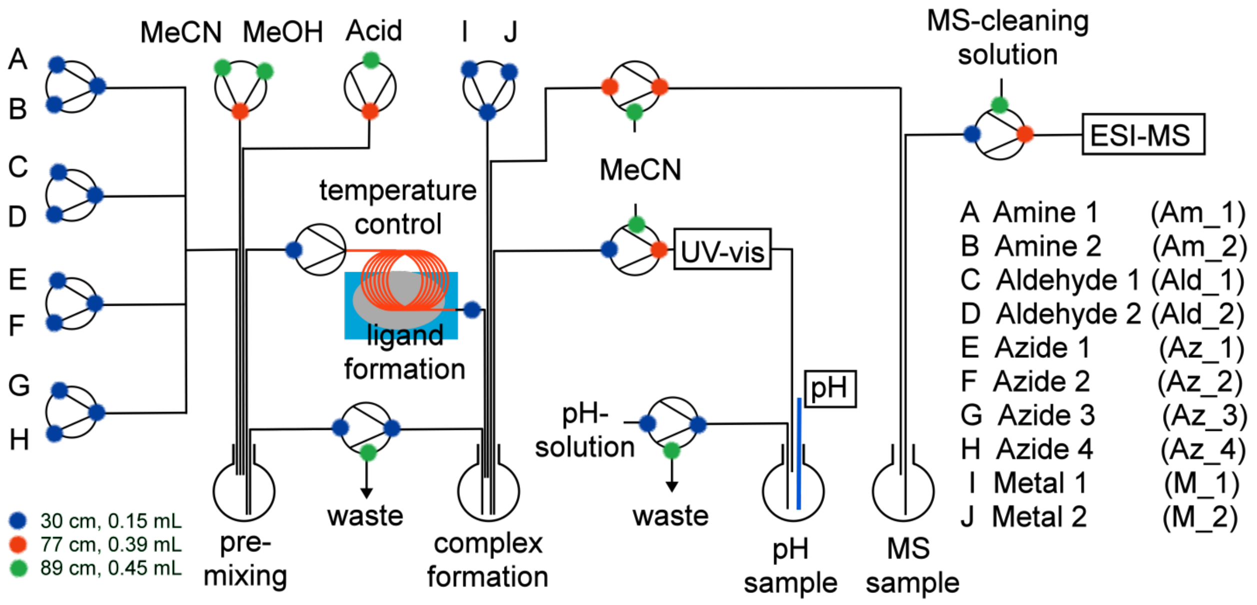

Figure S5 – Diagram showing the components and connections of the flow system.

Table S2 Hardware set-up.

**Number of pumps**	13
**Volume of the syringes**	5 mL
**Number of chambers**	5 (pre-mixing, ligand formation, complex formation, pH sample, MS sample)
**Reactor volume**	Ligand formation: 10.0 mL copper coil<br>Pre-mixing, complex formation: 10.0 mL RBF

	pH sample, MS sample: 5 mL RBF
**Reactor type**	Ligand formation: $^{1}/_{8}$ inch outer diameter copper tubing with an internal diameter of 1.6 mm and a tube length of 4.97 m<br>Pre-mixing, complex formation, pH sample, MS sample: round bottom flasks
**Connectors**	Standard connectors made of FPM and PEEK equipped with check valves (made of PEEK with a Chemraz® O-ring, which is compatible with organic solvents and compounds)
**In-line analysis**	ESI-MS, UV-vis, pH
**Control**	Alpha-Jump Exploration Algorithm (python, written bespoke for this project)

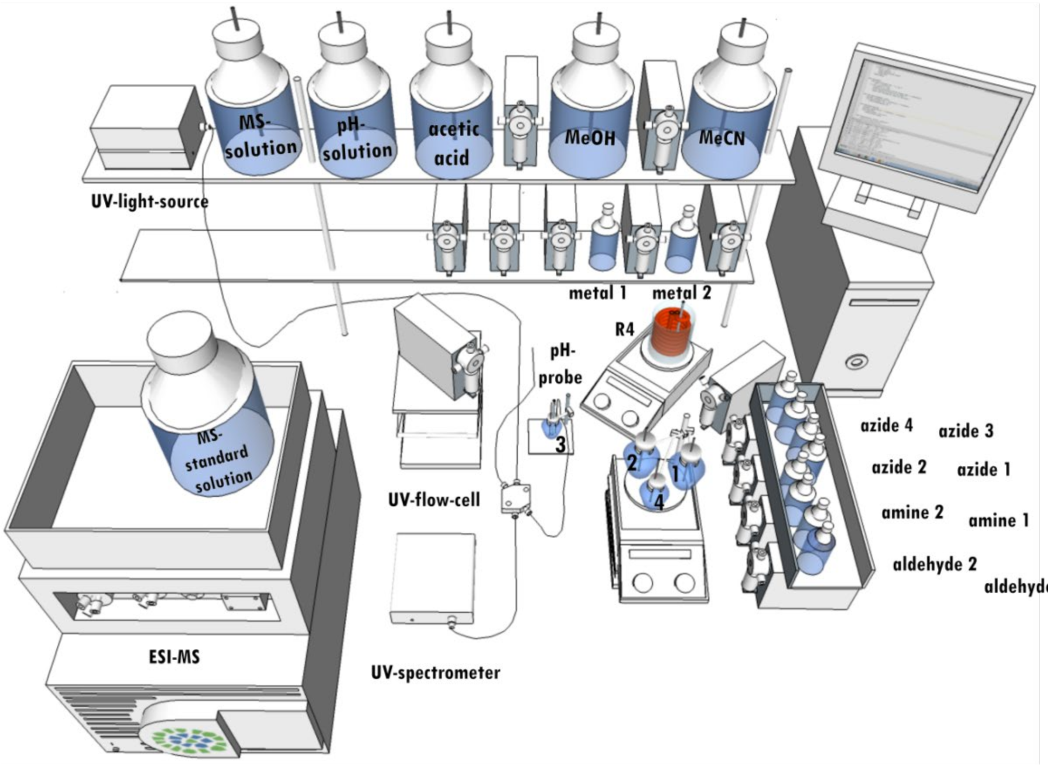

Figure S6 Schematic of the chemical robot consisting of a computer connected to 13 syringe pumps and three in-line analytical instruments – ESI-MS, UV-Vis flow cell and a pH probe. The solutions are moved into five different flasks during the experiment: the premixing flask (1) where the selected starting materials are mixed; the active copper reactor R4 (in red) where the ligand is formed; the complex formation reactor (2) where the metal solution is added to the ligand mixture; the dilution flask (3) where samples are prepared for UV-vis and pH measurements; and the ESI-MS flask (4) where samples are prepared for the spectrometric analysis. The tubing

connection between the pumps, reactors and the analytics are not displayed for clarity (see **Figure S5** for connectivity).

Five syringe pumps (5 mL) are connected to the ten possible reagent inputs (A-J, **Figure S5**) in the system, whilst the remaining eight 5 mL syringe pumps are used to move solvents and reaction mixtures between the reactors or the analytics. In particular, the organic reagents selected for the experiment are initially combined in the premixing reactor (1 in **Figure S6**) before being delivered to the activated flow reactor (in red, R4 in **Figure S6**), which is positioned on a computer-controlled hot-plate. The reaction solution from the activated reactor is transferred into a collection reactor (2 in **Figure S6**) where the solution can be sent to the analytical instruments or reacted with the metal salt solutions (I-J, **Figure S5**). The samples sent for pH-measurements pass through the UV-flow cell, at which time a measurement of the UV-vis spectrum is taken, before samples reach the dilution flask (3 in **Figure S6**). The sample solution is then disposed by direct transfer to the waste bottle. The samples sent for MS measurements are first placed in the corresponding MS preparation vial (4 in **Figure S6**) before transferring to the spectrometer. The sample analyzed by MS is also transferred directly to waste. The MS line is then cleaned using a solution of MeCN and H_2O as a 1/1 (*v*/*v*) mixture with 1% formic acid. One pump is associated with pure acetic acid, used for the activation of R4, whilst a second is linked to MeCN and MeOH used for the cleaning procedure before running a new experiment.

The chemical robot is intrinsically dependent on the connectivity of the liquid-handling system (the syringe pumps) with the reagents and the other components of the system. A configuration file with the overall connectivity allows our search engine to 'understand' this layout and autonomously direct the experiments.

3.1.1 Robot Operations

Once the experiment is chosen, the system is programmed to prime the syringe pumps (**Figure S7A**), set the hot plate temperature, and collect the UV-vis spectrum of the solvent to be used as a reference. Once the system has reached the selected reaction temperature, all reagents are mixed in the pre-mixing reactor (**Figure S7B**) before

transferring 10 mL of the solution into the chemical reactor at the selected temperature. If the total volume in the premixing vial is smaller than the volume of the copper coil reactor, MeCN will be added to reach 10 mL. If the total volume is greater than 10 mL any excess is directed to waste. After waiting for the stochastically selected reaction time, the reaction mixture is transferred from R4 to the complexation reactor (**Figure S7C**).

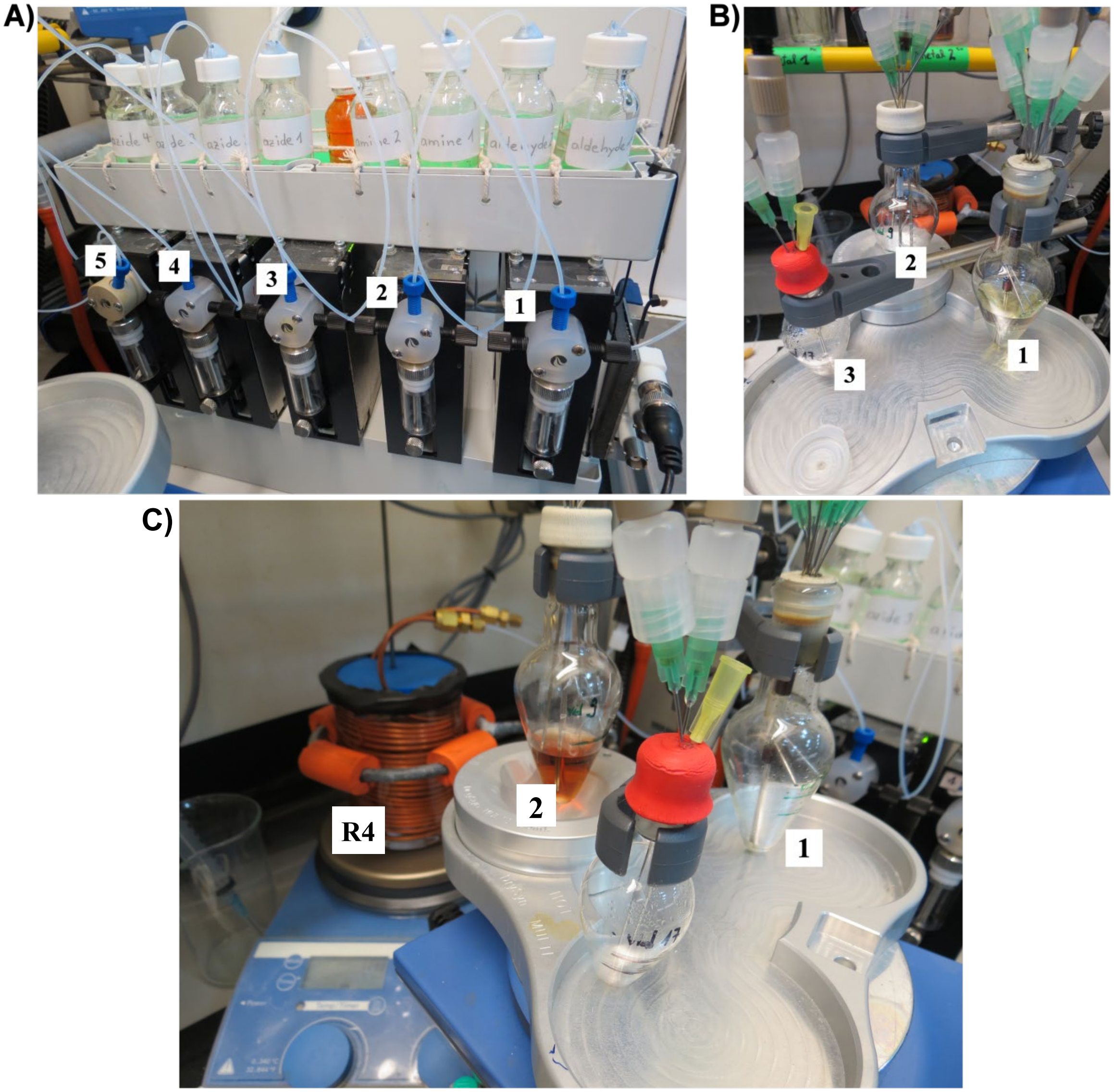

Figure S7 A) Photograph of the syringe pumps associated with the preparation of the ligand reaction mixture. **1** is the syringe pump associated with Ald_1 and Ald_2 solutions. **2** is the syringe pump associated with Am_1 and Am_2 solutions. **3** is the syringe pump associated with Az_1 and Az_2 solutions. **4** is the syringe pump associated with Az_3 and Az_4 solutions. **5** is the syringe pump used to move the reaction mixture from the premixing flask to R4. **B)** Photograph of the premixing flask (**1**), the complex formation flask (**2**), and the MS sample preparation flask (**3**). **C)** Photograph of the three flasks of the chemical robot including active reactor (**R4**). The

ligand mixture is collected as a reddish-brown solution (indicating complexation with Cu) in the complex formation flask (**2**) after the reaction time stochastically set in the experiment.

At this point the reaction mixture is analyzed (**Figure S8**). 1 mL of reaction mixture is moved from the complex formation flask to the MS preparation flask. From this flask, the sample is delivered to the benchtop MS using a dedicated syringe pump. 1.35 mL of reaction mixture is also separately sampled from the complex formation flask to the UV-vis flow cell, from where it is then directed to the dilution flask containing the pH probe.

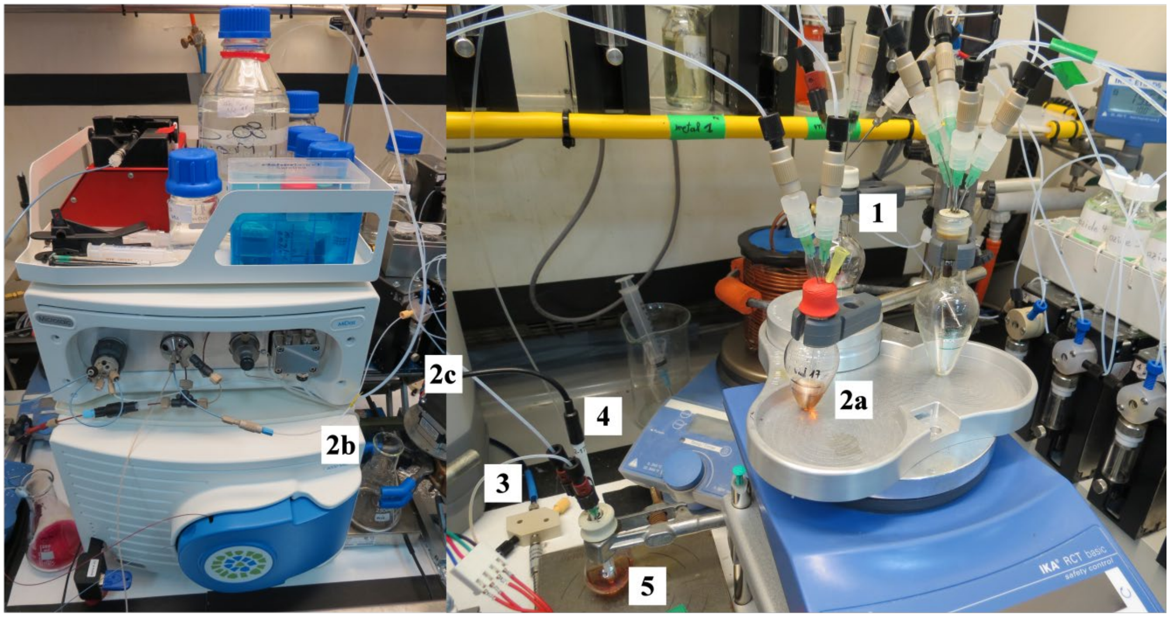

Figure S8 Photograph illustrating the infrastructure used in sample preparation for in-line analysis. The reaction mixture is sampled from the complex formation flask (**1**) to the MS preparation flask (**2a**). From this point the sample is delivered to the benchtop MS (**2b**) using a dedicated syringe pump (**2c**). The reaction mixture is also separately sampled from the complex formation flask (**1**) to the UV-Vis flow cell (**3**) and is then directed to the dilution flask (**5**) where the pH probe (**4**) is located.

The selected metal salt solution is then combined with the ligand mixture in the complex formation flask, giving an immediate noticeable colour change (**Figure S9B**). At this point an e-mail is sent to the operator to indicate that the complexation step is completed and the reaction solution can be physically collected if so desired (e.g. for crystallization). This pause in the procedure can also be bypassed if the operator decides to either not collect the samples or if the user is in the proximity of the system. After collecting the sample, the system cleans all parts of the robots which have been in contact with reagents and

reaction mixtures, including the plunger/syringe of the pump which potentially can choose between two different reagents, in order to limit contamination issues. At the same time, the chemical robot also autonomously calculates α for the selection of the next experiment, in order to start the preparation step (as previously described) as soon as the cleaning cycle is completed. If the α value is below an arbitrary threshold the system will email the operator and notify them of a possible point of interest.

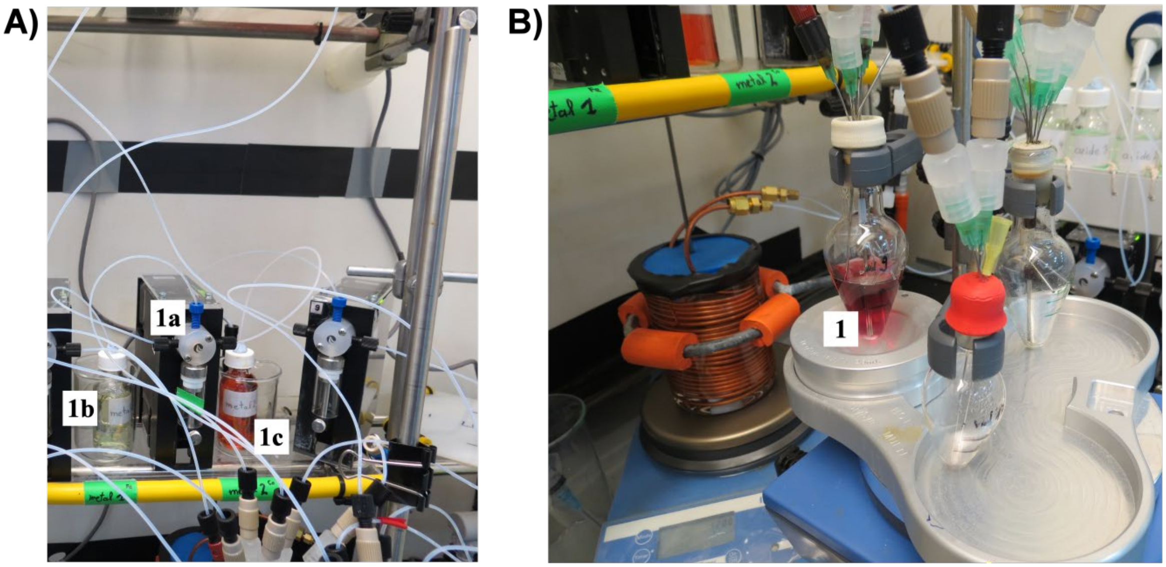

Figure S9 A) Photograph of the syringe pump (**1a**) used to deliver the two metal salts – $Fe(II)(ClO_4)_2$ (**1b**) and $Co(II)(ClO_4)_2$ (**1c**). **B)** Photograph of complexation flask (**1**) where the metal solutions are added to the ligand mixture resulting in a change of colour in the solution – from reddish-brown to purple in this specific case.

3.1.2 Temperature, Cross-Contamination and Reactor-Activation Control

The temperature can be changed from one experiment to another without the need for intervention as the hot plate in the system is computer-controlled. In order to better synchronize all chemical robot operations and have an estimation of each experiment duration (including waiting time), the time necessary to switch between temperatures was measured, as illustrated in **Figure S10**. The test was done in the temperature range of 30-80 °C (y axis). It was found that when heating from 30 °C to 80 °C (red bars), less than 20 min were required. However, cooling from 80 °C to 30 °C (blue bars) took more than one hour. To overcome the worst case scenario, cooling from 80 °C to 30 °C between one experiment and next, the system was programmed to set the temperature for the next

experiment as soon as the previous experiment is finished and before the cleaning procedure. The cleaning procedure takes 100 min during which all reactors, pumps and equipment are cleaned, and the copper tubing is activated. The cleaning of the tubing connected with the MS and the MS itself are also performed using a mixture of MeCN:H_2O (1:1, *v:v*) with 1% (*v/v*) of formic acid. The cleaning/activation procedure for the copper reactor consists of flushing the copper coil with different solvents. In particular, R4 is flushed with 10 mL of MeOH, before being filled with pure acetic acid. After 10 min, R4 is flushed with 10 mL of acetic acid first and afterwards with 20 mL of MeOH. R4 is filled with 10 mL of MeOH. After 10 min, R4 is flushed with 20 mL of MeOH first and afterwards with 20 mL of MeCN. R4 is then filled with 10 mL of MeCN. After 5 min, R4 is flushed with 50 mL of MeCN, before starting the next chosen reaction.

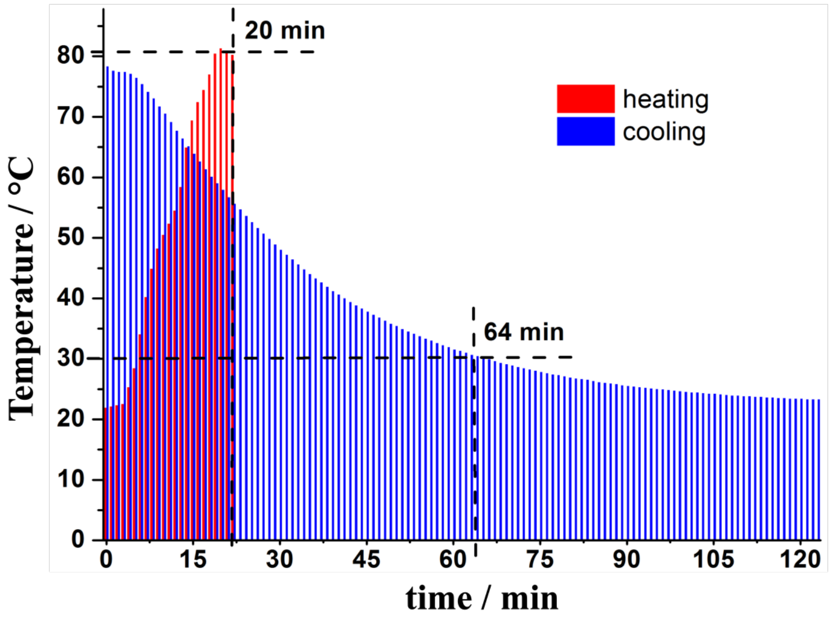

Figure S10 Diagram of temperature of the cooling and heating process *vs.* time.

3.1.3 Reliable automated acquisition of UV-vis data

The samples were diluted in order to have conclusive data that could be reliably processed by the chemical robot. The dilution step for both ligand and complex reaction mixtures was programmed to be performed in the syringe of a dedicated syringe pump. This pump was programmed to sample 1 mL of the reaction mixture to the UV-Vis flow

cell. After this first measurement, if the spectrum is saturated, another 0.35 mL of the reaction solution is withdrawn by the pump and mixed internally with MeCN to achieve a 1:10 dilution ratio. 0.35 mL of this diluted reaction mixture remains in the syringe, the rest is transferred to the UV-vis flow cell and the new spectrum is acquired. This dilution operation is repeated as needed on the remaining 0.35 mL until the measured spectrum has a maximum absorbance band smaller than 1 a.u. (**Figure S11**).

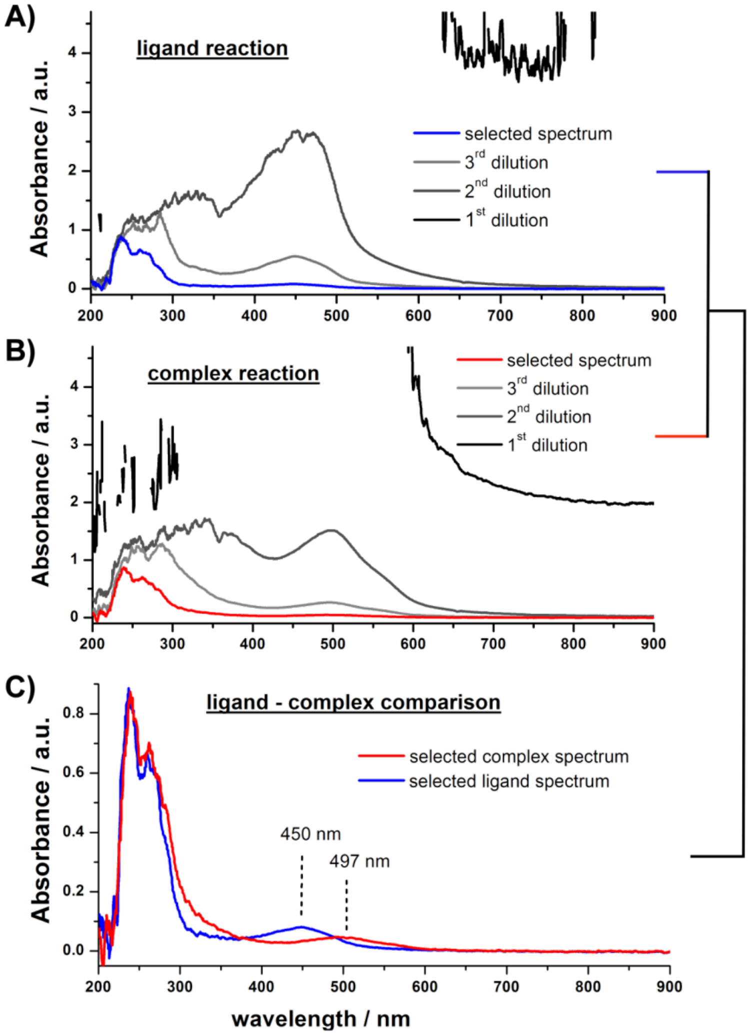

Figure S11 Dilution steps for the ligand reaction **A** (Ald1 4.1 mL, Am2 1.2, Az4 1.0 mL, 75 °C, 70 min), complex reaction **B** (addition of M2 1.5 mL) and a comparison of the automatically selected UV-vis spectra of both steps.

3.1.4 Automated acquisition of pH-values

A sample of reaction mixture in MeCN is mixed with an excess of MeCN:H_2O 1:1 (*v*/*v*), referred to as pH_solution before the measurements. We also set a suitable cleaning procedure for the probe, by checking the pH_solution value after each measurement. 0.25 mL of the reaction solution was diluted with 3 mL of pH_solution, so the liquid covers the active region of the probe. We could clearly observe changes in the pH values among the different classes of starting materials. In particular the pH range of aldehyde is 4-5, of amines is 8-9, of azides is 6-7 and of metals is 2-4.

3.1.5 Example measurements for a single experiment

Figure S12 shows the analytical output from the set of three instruments on the same experiment.

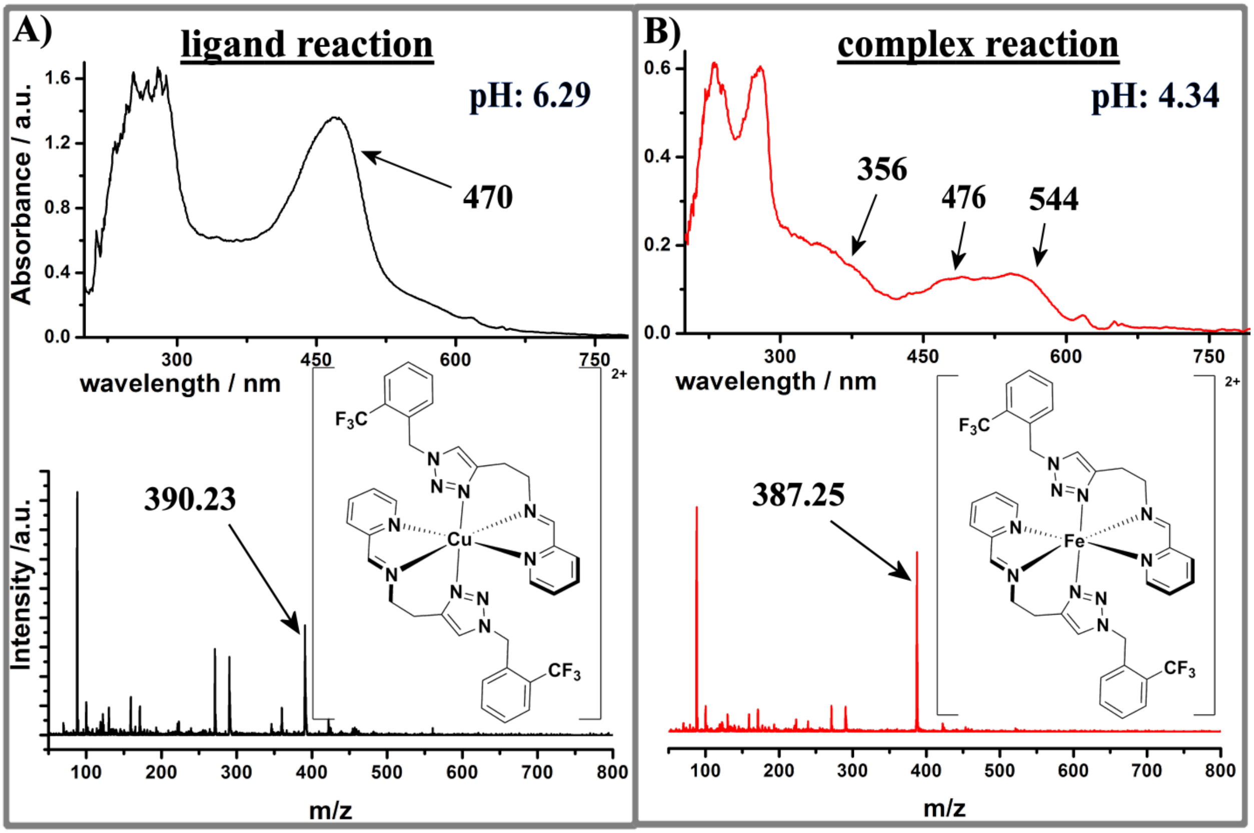

Figure S12 The MS, UV-vis and pH data for the ligand formation reaction (Ald2 0.5mL, Am2 4.7mL. Az1 4.7mL, 60°C, 50 min) is displayed in **A).** The subsequent addition of 0.8 mL of M2 causes the complex formation reaction to take place and the measurements of which are in **B)**.

3.2 SOFTWARE

3.2.1 Connections and Communication

The physical connection between the pumps and the computer was built based on wiring RS232 cables to a daisy chain assembly. The protocol used to build the command scripts and to communicate between the PC and the pumps is originally created by the pump manufacturer. A Custom-made developed Python™ code was employed to program the pumps to deliver the desired flow-rates and to control the in-line analytics using the property programming language. The Python™-based PC interface and protocols developed using the API provided by the property company of all computer controllable piece of equipment in the robot – 13 Tricontinent syringe pumps, a Microaic ESI-MS, a UV-vis flow cell, a pH probe and a hot plate.

3.2.2 Non-deterministic Routing

The platform uses a form of non-deterministic routing to perform the required liquid-handling operations. Standard liquid handling systems usually assign specific routes from the different operations that need to be undertaken so a movement of a solution from any given staring point to an end point would be executed in only one defined way. In non-deterministic routing the path is not statically set. The system holds in memory a bipartite graph representation of its configuration. The vials holding the different materials are one type of node in the graph and so are the destination points such as analytical instruments, reactors and waste. The second type of node is the valve and the edges are the connections between the nodes. Each part of the graph, be it node or edge, has associated attributes with it. For instance a valve node contains information about its type, address and other technical information, a container node has information about what material it contains, the current volume and so on. The attributes of all the parts are changed automatically during operation. As an example if a solution is moved from one vial to another then that volume is subtracted from the volume attribute of the former and added to the latter. In the case when a volume needs to be moved that exceeds the

volume of available material an error message is displayed and logged. An important attribute that all parts of the graph have is whether each part is clean or dirty. Holding any material or having material pass through a part of the system will mark it as dirty. Following a cleaning procedure the part will be marked as clean.

The system operates by making a stochastic decision each time it needs to transfer material. It uses standard graph algorithms to choose the shortest path that has no unclean points in its way. **Figure S13** shows several cases on an example graph representation. Part A shows a case where there are two equidistant paths to get from point E to point G. The shortest path finding algorithm will return a single path selected at random out of all such paths that are of the same shortest length. In this case it will have equal probability of choosing either the red or the green path. In part B there is only one shortest path between points B and E, the pink path. Following this operation, the path between B and E is now unclean and so when the system needs to move from point G to point E, part C of the figure, it will only have the orange path to take since it bypasses the dirty sections.

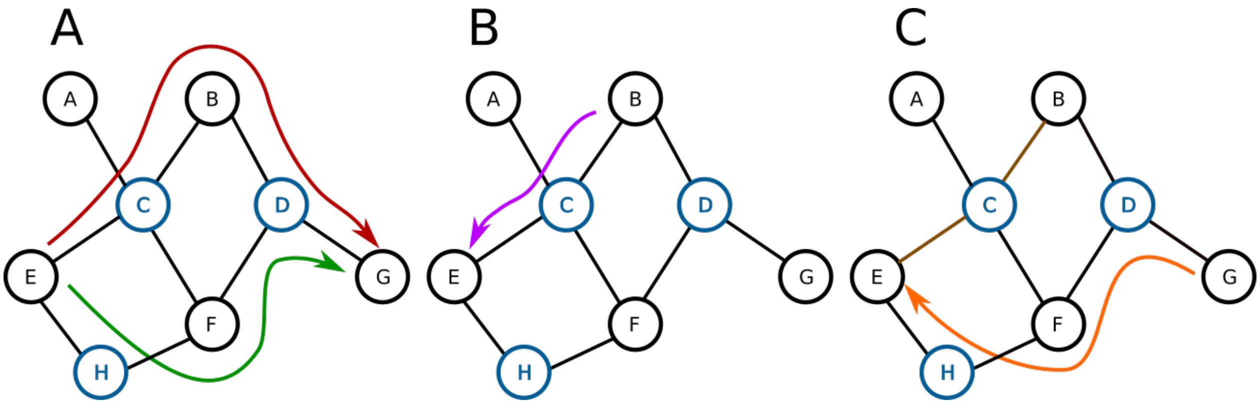

Figure S13 – A diagram depicting the connections in a system as a network graph. Nodes depict either locations, in black, or valves, in blue, and the edges signify the physical connections between the nodes. Subplot A shows two equivalent paths, in red and green, to get from node E to node G, the system will randomly choose one of them. Subplot B shows the shortest path to get from node B to node E. In subplot C the connections between nodes E and C, and C and B are not clean and therefore the system chooses the show path, in orange, to get from node G to node E.

By using a non-deterministic path choosing system we gain more flexibility for the same physical configuration than with a deterministic system. For example, in our platform the number of connections possible using defined paths would be 171 but when using a stochastic system the number of possible paths rises by an order of magnitude to 1128. The increased versatility is especially useful in dynamic system where the non-deterministic element allows the system to adapt to changing conditions in a flexible manner without the need of rigid specific definitions. Without making any assumptions about the directionality within the graph the number of possible pathways increases to 3262.

4 SYNTHESES

4.1 AZIDE SYNTHESES

General Procedure: Azides were synthesized by variations of published methods.[7-9] Added NaN_3 (1.706 g, 26.25 mmol) to a stirred solution of the corresponding bromide (17.5 mmol) in a 50 mL water/acetone mixture (*v*/*v*: 1/4). The resulting suspension was stirred at room temperature for three days. DCM was added to the mixture and the organic layer was separated. The aqueous layer was extracted with DCM three times. The combined organic phase was dried over $MgSO_4$. Solvent was removed under reduced pressure, and the azide was sufficiently pure to use without further work-up (>99%). Further purification can be achieved by column chromatography using a mixture of EtOAc and PE (*v*/*v* : 5/1) yielding the pure azide. Conversions below were calculated by ^{1}H-NMR.

4-trifluoromethylbenzylazide

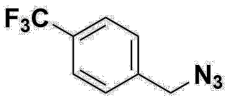

Crude yield: 3.19 g (78.7%). **^{1}H-NMR:** ($CDCl_3$, 400 MHz): δ = 7.65 (d, ^3J = 8.1 *Hz*, 2H, C*H*Ar), 7.44 (d, ^3J = 8.1 *Hz*, 2H, C*H*Ar), 4.42 (s, 2H, CH_2) ppm. **GC-MS** (m/z): 201.1 ($[M]^+$, *t* = 11.1 min)

2-trifluoromethylbenzylazide

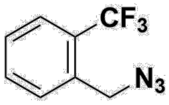

Crude yield: 3.38 g (83.7%) **^{1}H-NMR:** ($CDCl_3$, 400 MHz): δ = 7.71-7.69 (m, 1H, C*H*Ar), 7.62-7.57 (m, 2H, C*H*Ar), 7.47-7.43 (m, 1H, C*H*Ar), 4.58 (s, 2H, CH_2) ppm. **GC-MS** (m/z): 201.1 ($[M]^+$, *t* = 10.2 min)

1,4-bis-(azidomethyl)benzene

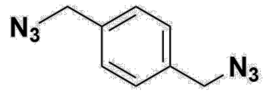

Crude yields: 3.37 g (89.4%) **^{1}H-NMR:** ($CDCl_3$, 400 MHz): δ = 7.34 (s, 4H, C*H*Ar), 4.35 (s, 4H, CH_2) ppm. **^{13}C-NMR:** ($CDCl_3$, 100 MHz): δ = 135.4 (C_qAr), 128.4 (CHAr), 54.1 (CH_2) ppm. **GC-MS** (m/z): 188.1 ($[M]^+$, *t* = 20.3 min)

1,3,5-tris-(azidomethyl)benzene

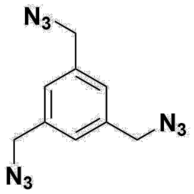

To a stirred solution of 1,3,5-*tris*-(bromomethyl)benzene (2.0 g, 5.6 mmol) in 250 mL DMSO was added NaN_3 (0.771 g, 14.0 mmol). The resulting suspension was stirred at room temperature for five days and then quenched with water (600 mL). DCM was added to the mixture and the organic layer was separated. The aqueous layer was extracted three times with DCM. The combined organic phase was washed with brine and dried over $MgSO_4$. Solvent was removed under reduced pressure, and the azide was sufficiently pure to use without further work-up.

Crude yield: 1.08 g (79.6%) **^{1}H-NMR:** (DMSO, 400 MHz): δ = 7.36 (s, 3H, C*H*Ar), 4.53 (s, 6H, CH_2) ppm. **^{13}C-NMR:** (DMSO, 100 MHz): δ = 136.8 (C_qAr), 126.8 (CHAr), 53.1 (CH_2) ppm. **GC-MS** (m/z): 243.1 ($[M]^+$, *t* = 27.7 min)

4.2 LIGAND VARIETY

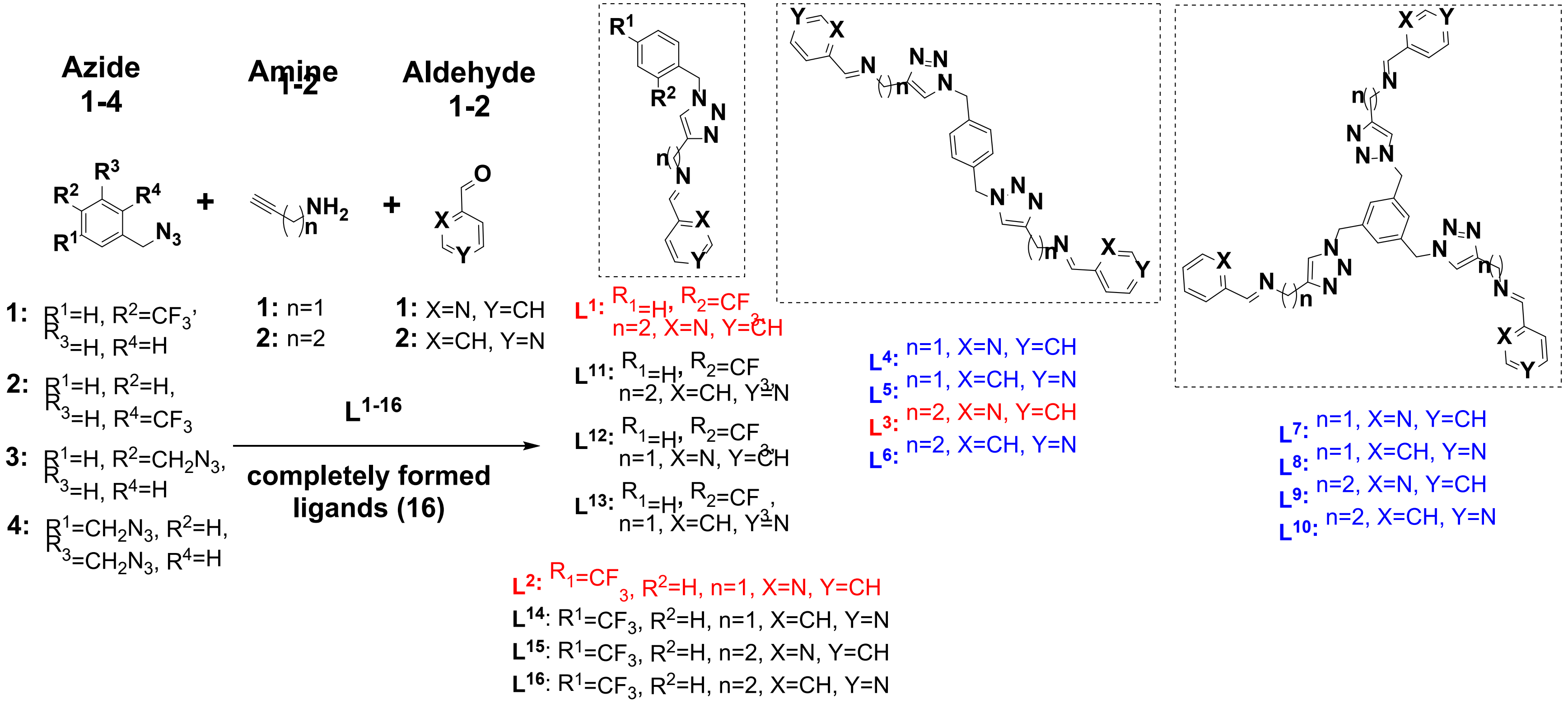

L^{1-3}: ligands fully formed and lead to isolated complex formations

L^{4-10}: ligands fully formed: used to prove the reliability and efficiency with the copper reactor

L^{11-16}: other possible constitutions of the full ligand design

Figure S14 – Scheme showing the starting materials and all possible ways to construct ligands assuming complete reactions.

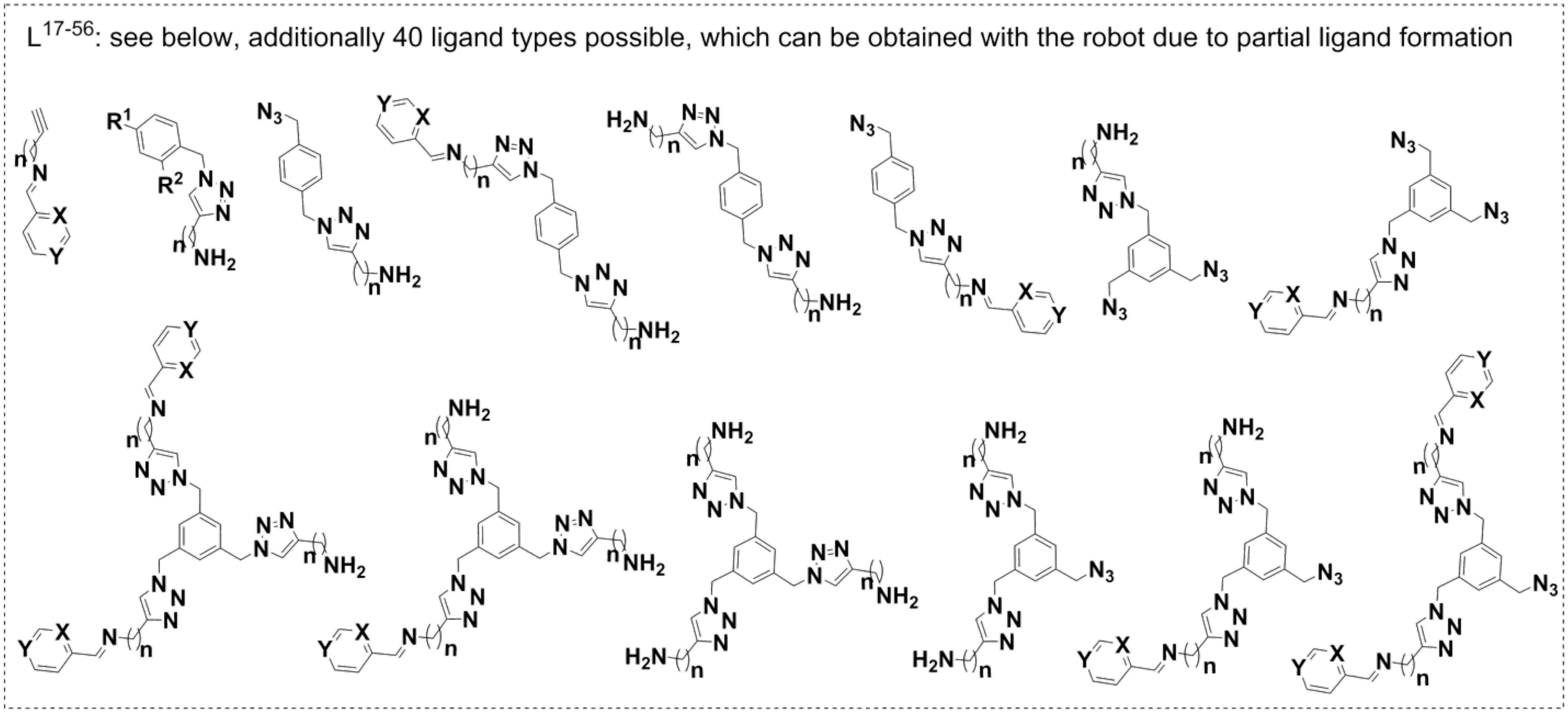

Figure S15 – Scheme indicating possible ways to construct ligands with partial reactions.

4.3 SELECTED LIGAND SYNTHESES

General activation procedure: the syntheses reported were all performed after activation of the Cu tubing (R4) using acetic acid. This procedure consists of flushing R4 (heated at 80 °C) with different solvents. Firstly, R4 is filled with MeOH that is immediately pushed out by acetic acid. After 10 min, acetic acid is pushed out from R4 using MeOH. After 10 min, MeOH is pushed out using MeCN that remains in the copper reactor for 5 min. R4 is finally flushed with MeCN another five times before starting the chosen reaction.

Synthesis procedure: The chosen azide and aminoalkyne (2.5 mL of each in MeCN, See **Table S3** for concentrations) were combined in one flask and the resulting mixture was introduced into R4 together with a solution of the selected aldehyde (5 mL in MeCN) at the same flow rate of 0.1 mL min^{-1}. After 50 min at 80 °C, the solution was collected at the copper coil outlet and subsequently analyzed off-line.

Selected Isolated Ligands:

***N*-[(pyridine-2-yl)-methyliden]-*C*-[1-(2-trifluoromethylbenzyl)-1*H*-1,2,3-triazol-4-yl]-ethylamine (L^{1})**

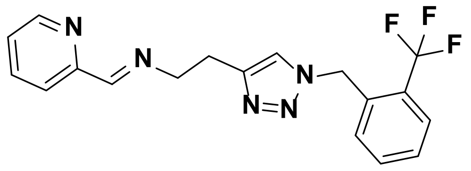

^{1}H-NMR: (CD_3CN, 400 MHz): δ = 10.01 (d, *J* = 0.7 Hz, 2H, C*H*N), 8.79 (d, *J* = 4.7 Hz, 2H, C*H*Ar), 8.59 (br. s, 4H, C*H*Ar), 8.24 (br. s, 4H, C*H*Ar), 7.92 (ddd, J = 7.8, 1.7, 1.0 Hz, 2H, C*H*Ar), 7.79 (s, 2H, C*H*triazole), 7.72 (d, *J* = 3.8 Hz, 2H, C*H*Ar), 7.61 – 7.58 (m, 2H, C*H*Ar), 7.48 (dd, *J* = 6.4, 2.6 Hz, 2H, C*H*Ar), 7.03 – 6.89 (m, 2H, C*H*Ar), 5.67 (s, 4H, CH_2), 4.15 – 3.68 (br. s, 4H, CH_2), 3.27 – 2.81 (br. s, 4H, CH_2) ppm. **^{13}C-NMR:** (CD_3CN, 100 MHz): δ = 194.7 (*C*HAr), 154.0 (CHAr), 151.4 (CHAr), 138.4 (CHAr), 137.6 (CHAr), 135.0 (CHAr), 133.9 (CHAr), 130.8 (CHAr), 129.7 (CHAr), 127.2 (q, *J* = 5.7 Hz, CF_3), 50.9 (br. s, CH_2). ^{19}F NMR (CD_3CN, 376 MHz) δ = -59.97 (s). **ESI-MS:** *m/z* 360.11 [**L^{1}**+H]$^{+}$, 390.23 [Cu(**L^{1}**)$_2$]$^{2+}$. **UV-vis:** (MeCN) λ_{max}, nm: 470. **pH of reaction mixture:** 6.29.

***N*-[(pyridine-2-yl)-methyliden]-*C*-[1-(4-trifluoromethylbenzyl)-1*H*-1,2,3-triazol-4-yl]-methylamine (L^{2})**

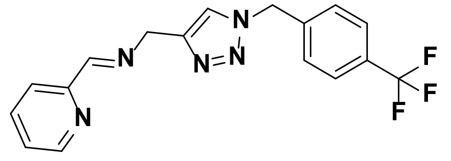

^{1}H-NMR: (CD_3CN, 400 MHz): δ = 8.49 (s, 1H, C*H*N), 8.65 (td, ^{3}Jd = 4.8 *Hz*, ^{4}Jt = 1.1 *Hz*, 1H, C*H*Ar), 7.98 (td, ^{3}Jd = 7.8 *Hz*, ^{4}Jt = 1.1 *Hz*, 1H, C*H*Ar), 7.81 (dt, ^{3}Jt = 7.8 *Hz*, ^{4}Jd = 1.1 *Hz*, 1H, C*H*Ar), 7.79 (s, 1H, C*H*triazole), 7.71 (d, ^{3}J = 8.1 *Hz*, 2H, C*H*Ar), 7.48 (d, ^{3}J = 8.1 *Hz*, 2H, C*H*Ar), 7.41 (ddd, ^{3}J = 7.8 *Hz*, ^{3}J = 4.8 *Hz*, ^{4}J = 1.1 *Hz*, 1H, C*H*Ar), 5.65 (s, 2H, CH_2), 4.92 5.42 (s, 2H, CH_2) ppm. **^{13}C-NMR:** (CD_3CN, 100 MHz): δ = 164.4 (CHN), 155.1 (C_qAr), 150.1 (C_qAr), 146.4 (C_qAr), 137.3 (CHAr), 129.1 (CHAr), 126.4 (CHAr), 126.2 (C_qAr), 125.7 (CHAr), 123.5 (CHAr), 121.2 (CHAr), 55.8 (CH_2), 53.4 (CH_2) ppm. **Benchtop ESI-MS:** *m/z* 346.14 [**L^{2}**+H]$^{+}$, 408.37 [**L^{2}**+Cu]$^{+}$.

Pyridin-2-ylmethylene-(2-{1-[4-(4-{2-[(pyridin-2-ylmethylene)-amino]-ethyl}-[1,2,3]triazol-1-ylmethyl)-benzyl]-1*H*-[1,2,3]triazol-4-yl}-ethyl)-amine (L^3)

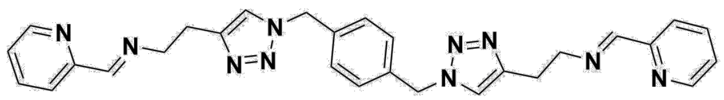

This compound was synthesized using the **synthesis procedure**, by combining 3-butynylamine (69 mg, 1 mmol), 1,4-*bis*-(azidomethyl)benzene (94.04 mg, 0.5 mmol) and 2-pyridinecarboxaldehyde (107 mg, 1 mmol) prepared as 0.4, 0.2 and 0.2 M solutions in MeCN.

Conversion yield: 61.5% **^{1}H-NMR (400 MHz):** (DMSO, 400 MHz): δ = 8.63 (bb, 2H, C*H*Ar), 8.29 (bb, 2H, C*H*N), 7.89 (bb, 6H, C*H*Ar), 7.49 (bb, 2H, C*H*Ar), 7.14 (bb, 4H, C*H*Ar), 5.52 (bb, 4H, CH_2), 3.90 (bb, 4H, CH_2), 3.00 (bb, 4H, CH_2) ppm. **^{1}H NMR (600 MHz):** (DMSO, 600 MHz) δ = 8.61 (br s, 2H, Py-*H*), 8.29 (br s, 2H, RN=C*H*R), 8.00-7.78 (m, 6H, Py-*H*, Py-*H*, Triazole-*H*), 7.47 (br s, 2H, Py-*H*), 7.13 (s, 4H, Ar-*H*), 5.51 (s, 4H, Ar-C*H*2-Triazole), 3.91 (br s, 4H, R-CH2-N=C*H*R), 2.99 (br s, 4H, Triazole-C*H*2-R) ppm. **ESI-MS:** *m/z* 505.3 [$\mathbf{L^3}$+H]$^+$, 567.2 [$\mathbf{L^3}$+Cu]$^+$, 535.7 [$\mathbf{2L^3}$+Cu]$^{2+}$, 787.8 [$\mathbf{3L^3}$+Cu]$^{2+}$

Pyridin-2-ylmethylene-{1-[4-(4-{[(pyridin-2-ylmethylene)-amino]-methyl}-[1,2,3]triazol-1-ylmethyl)-benzyl]-1*H*-[1,2,3]triazol-4-ylmethyl}-amine (L[4])

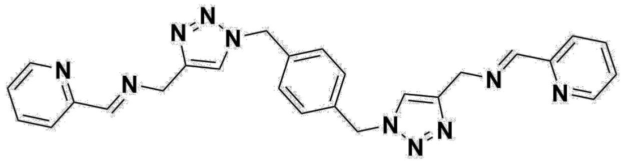

This compound was synthesized using the **synthesis procedure**, by combining propargylamine (55 mg, 1 mmol), 1,4-*bis*-(azidomethyl)benzene (94.04 mg, 0.5 mmol) and 2-pyridinecarboxaldehyde (107 mg, 1 mmol) prepared as 0.4 M, 0.2 M and 0.2 M solutions in MeCN.

Conversion: 81.3% **^{1}H-NMR:** (CD_3CN, 400 MHz): δ = 8.12-8.11 (m, 4H, C*H*Ar), 7.97 (s, 2H, C*H*N), 7.53 (s, 2H, C*H*triazole), 7.13-7.12 (m, 4H, C*H*Ar), 6.77 (s, 4H, C*H*Ar), 5.00 (s, 4H, C*H*$_2$), 4.30 (s, 4H, C*H*$_2$) ppm. **^{13}C-NMR:** ($CDCl_3$, 100 MHz): δ = 161.3 (CHN), 150.2 (CHAr), 144.7 (C_qAr), 142.3 (C_qAr), 135.9 (C_qAr), 128.3 (CHAr), 123.2 (CHAr), 121.8 (CHAr), 55.0 (CH_2) 52.3 (CH_2) ppm. **ESI-MS:** *m/z* 476.22 [**L^4**+H]$^+$, 539.15 [**L^4**+Cu]$^+$.

Pyridin-2-ylmethylene-{1-[4-(4-{[(pyridin-4-ylmethylene)-amino]-methyl}-[1,2,3]triazol-1-ylmethyl)-benzyl]-1*H*-[1,2,3]triazol-4-ylmethyl}-amine (L[5])

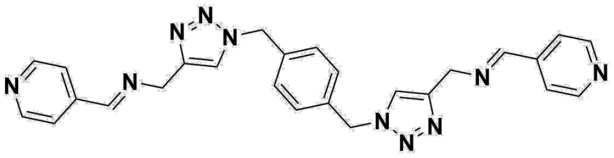

This compound was synthesized using the **synthesis procedure**, by combining propargylamine (55 mg, 1 mmol), 1,4-*bis*-(azidomethyl)benzene (94.04 mg, 0.5 mmol) and 4-pyridinecarboxaldehyde (107 mg, 1 mmol) prepared as 0.4, 0.2 and 0.2 M solutions in MeCN.

Conversion: 65.4% **^{1}H-NMR:** (DMSO, 400 MHz): δ = 8.79 (bb, 4H, C*H*Ar), 8.51 (s, 2H, C*H*N), 8.83 (s, 2H, C*H*triazole), 7.67 (d, ^2J = 5.3 *Hz*), 4H, CHAr), 7.35 (s, 4H, CHAr), 5.58 (s, 4H, C*H*$_2$), 4.92 (bb, 4H, C*H*$_2$) ppm. **^{13}C-NMR:** (DMSO, 100 MHz): δ = 161.4 (CHN), 150.3 (CHAr), 144.7 (C_qAr), 142.4 (C_qAr), 136.1 (C_qAr), 128.4 (CHAr), 123.2 (CHAr), 121.8 (CHAr), 55.1 (CH_2) 52.4 (CH_2) ppm.

Pyridin-2-ylmethylene-(2-{1-[4-(4-{2-[(pyridin-4-ylmethylene)-amino]-ethyl}-[1,2,3]triazol-1-ylmethyl)-benzyl]-1*H*-[1,2,3]triazol-4-yl}-ethyl)-amine (L^{6})

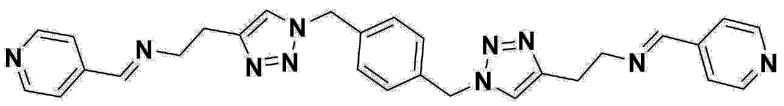

This compound was synthesized using the **synthesis procedure**, by combining 3-butynylamine (69 mg, 1 mmol), 1,4-*bis*-(azidomethyl)benzene (94.04 mg, 0.5 mmol in 2.5 mL of MeCN) and 4-pyridinecarboxaldehyde (107 mg, 1 mmol in 5 mL of MeCN) prepared as 0.4, 0.2 and 0.2 M solutions in MeCN.

Conversion yield: 80.9% **^{1}H-NMR:** (DMSO, 400 MHz): δ = 8.69 (bb, 4H, C*H*Ar), 8.29 (s, 2H, C*H*N), 7.89 (s, 2H, C*H*triazole), 7.61 (bb, 4H, C*H*Ar), 7.13 (bb, 4H, C*H*Ar), 5.50 (s, 4H, CH_2), 3.86 (t, 4H, *J* = 7.0 *Hz*, CH_2), 2.97 (t, 4H, *J* = 7.0 *Hz*, CH_2) ppm. **^{13}C-NMR:** (DMSO, 100 MHz): δ = 160.2 (CHN), 150.3 (CHAr), 144.9 (C_qAr), 142.4 (C_qAr), 136.0 (C_qAr), 128.1 (CHAr), 122.8 (CHAr), 121.8 (CHAr), 59.8 (CH_2), 52.2 (CH_2), 26.6 (CH_2) ppm. **ESI-MS:** *m/z* 504.2498 [**L^{6}**]$^{+}$, 567.17 [**L^{6}**+Cu]$^{+}$.

{1-[3,5-Bis-(4-{[(pyridin-2-ylmethylene)-amino]-methyl}-[1,2,3]triazol-1-ylmethyl)-benzyl]-1*H*-[1,2,3]triazol-4-ylmethyl}-pyridin-2-ylmethylene-amine (L^7)

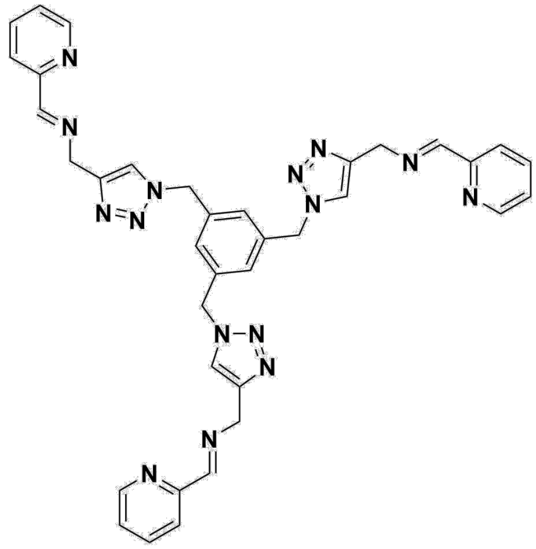

This compound was synthesized using the **synthesis procedure**, by combining propargylamine (83 mg, 1.5 mmol), 1,3,5-*tris*-(azidomethyl)benzene (122 mg, 0.5 mmol) and 2-pyridinecarboxaldehyde (161 mg, 1.5 mmol) prepared as 0.6, 0.2 and 0.3 M solutions in MeCN.

Conversion yield: 79.3% **^{1}H-NMR:** (DMSO, 400 MHz): δ = 8.64-8.63 (m, 3H, C*H*Ar), 8.45 (s, 3H, C*H*N), 8.05 (s, 3H, C*H*triazole), 7.95-7.92 (m, 3H, C*H*Ar), 7.85-7.84 (m, 3H, C*H*Ar), 7.47-7.44 (m, 3H, C*H*Ar), 7.26 (s, 3H, C*H*Ar), 5.56 (s, 6H, CH_2), 4.87 (s, 6H, CH_2) ppm. **^{13}C-NMR:** (DMSO, 100 MHz): δ = 163.5 (CHN), 153.9 (C_qAr), 149.2 (CHAr), 144.8 (CHAr), 137.7 (C_qAr), 137.3 (C_qAr), 127.8 (CHAr), 125.3 (CHAr), 123.4 (CHAr), 120.6 (CHAr), 54.7 (CH_2), 52.3 (CH_2) ppm. **ESI-MS:** *m/z* 369.1143 [**L**^{7}+Cu]$^{2+}$, 698.288 [**L**^{7}+Na]$^+$.

{1-[3,5-Bis-(4-{[(pyridin-4-ylmethylene)-amino]-methyl}-[1,2,3]triazol-1-ylmethyl)-benzyl]-1*H*-[1,2,3]triazol-4-ylmethyl}-pyridin-2-ylmethylene-amine (L^8)

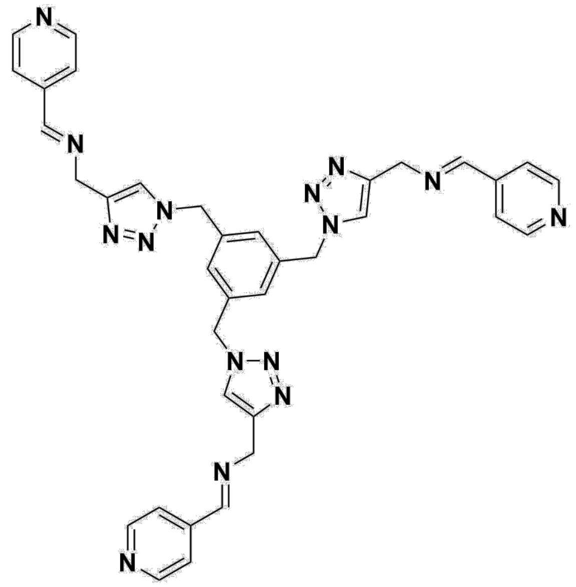

This compound was synthesized using the **synthesis procedure**, by combining propargylamine (83 mg, 1.5 mmol), 1,3,5-*tris*-(azidomethyl)benzene (122 mg, 0.5 mmol) and 4-pyridinecarboxaldehyde (161 mg, 1.5 mmol) prepared as 0.6, 0.2 and 0.3 M solutions in MeCN.

Conversion yields: 80.0% **^{1}H-NMR:** (DMSO, 400 MHz): δ = 8.68 (bb, 6H, C*H*Ar), 8.50 (s, 3H, C*H*N), 8.06 (s, 3H, C*H*triazole), 7.68 (bb, 6H, C*H*Ar), 7.27 (s, 3H, C*H*Ar), 5.56 (s, 6H, CH_2), 4.85 (s, 6H, CH_2) ppm. **^{13}C-NMR:** (DMSO, 100 MHz): δ = 161.5 (CHN), 150.4 (C_qAr), 137.4 (C_qAr), 137.0 (C_qAr), 127.9 (CHAr), 127.3 (CHAr), 123.4 (CHAr), 122.0 (CHAr), 55.1 (CH_2), 53.1 (CH_2) ppm.

(2-{1-[3,5-Bis-(4-{2-[(pyridin-2-ylmethylene)-amino]-ethyl}-[1,2,3]triazol-1-ylmethyl)-benzyl]-1*H*-[1,2,3]triazol-4-yl}-ethyl)-pyridin-2-ylmethylene-amine (L[9])

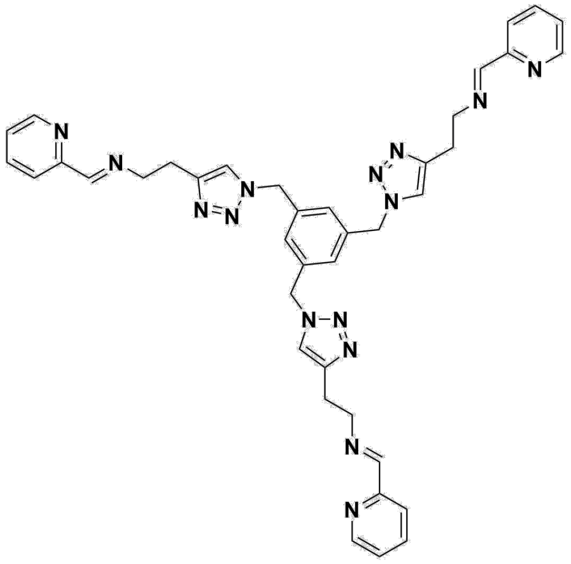

This compound was synthesized using the **synthesis procedure**, by combining 3-butynylamine (102 mg, 1.5 mmol), 1,3,5-*tris*-(azidomethyl)benzene (122 mg, 0.5 mmol) and 2-pyridinecarboxaldehyde (161 mg, 1.5 mmol) prepared as 0.6, 0.2 and 0.3 M solutions in MeCN.

Conversion yield: 80.9% ^{1}**H-NMR:** (DMSO, 400 MHz): δ = 8.61 (bb, 3H, C*H*Ar), 8.32 (bb, 3H, C*H*N), 7.96-782 (m, 12H, C*H*Ar), 7.10 (s, 3H, C*H*Ar), 5.49 (s, 6H, CH_2), 3.90 (bb, 6H, CH_2), 3.00 (bb, 6H, CH_2) ppm. ^{13}**C-NMR:** (DMSO, 100 MHz): δ = 162.4 (CHN), 154.0 (C_qAr), 149.4 (CHAr), 145.4 (CHAr), 137.4 (C_qAr), 136.8 (C_qAr), 127.9 (CHAr), 127.7 (CHAr), 126.8 (CHAr), 125.2 (CHAr), 122.7 (CHAr), 120.5 (CHAr), 59.7 (CH_2), 52.1 (CH_2), 27.0 (CH_2) ppm. **ESI-MS:** *m/z* 390.1385 [**L^{9}**+Cu]$^{2+}$, 740.3293 [**L^{9}**+Na]$^{+}$.

(2-{1-[3,5-Bis-(4-{2-[(pyridin-4-ylmethylene)-amino]-ethyl}-[1,2,3]triazol-1-ylmethyl)-benzyl]-1*H*-[1,2,3]triazol-4-yl}-ethyl)-pyridin-2-ylmethylene-amine (L^{10})

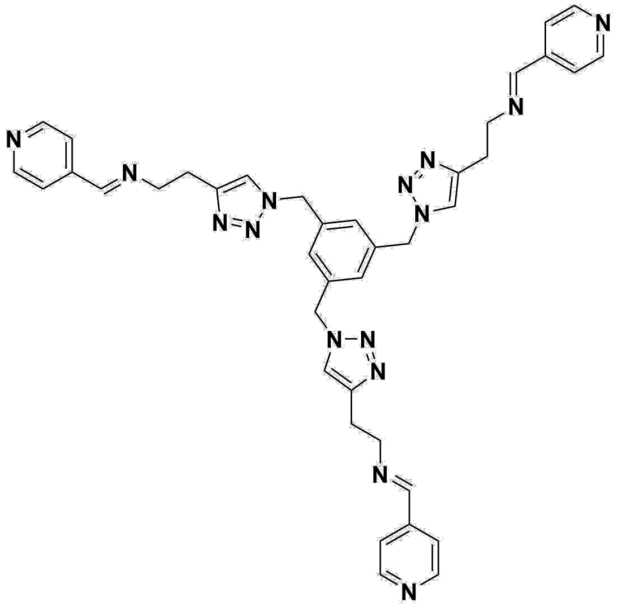

This compound was synthesized using the **synthesis procedure**, by combining 3-butynylamine (102 mg, 1.5 mmol), 1,3,5-*tris*-(azidomethyl)benzene (122 mg, 0.5 mmol) and 4-pyridinecarboxaldehyde (161 mg, 1.5 mmol) prepared as 0.6, 0.2 and 0.3 M solutions in MeCN.

Conversion yield: 86.8% **^{1}H-NMR:** (DMSO, 400 MHz): δ = 8.65 (bb, 6H, C*H*Ar), 8.33 (s, 3H, C*H*N), 7.89 (s, 3H, C*H*triazole), 7.62 (bb, 6H, C*H*Ar), 7.13 (s, 3H, C*H*Ar), 5.47 (s, 6H, C*H*$_2$), 3.87 (t, *J* = 7.1 *Hz*, 6H, C*H*$_2$), 2.98 (t, *J* = 7.1 *Hz*, 6H, C*H*$_2$) ppm.

4.4 COMPLEXES DISCOVERED AUTONOMOUSLY

Most of the products of the ligand reaction were not isolated due to the approach used in exploration, which does not include purification. Therefore, in most of the cases, the crude reaction mixtures were analysed to identify whether any reaction had proceeded. Analysis of the crude reaction mixtures allowed for assignment of ^{1}H- and ^{13}C-NMR signals. All starting materials were used as solutions in acetonitrile, with concentration as reported in **Table S3**. The reaction solutions collected for crystallization are in the range of 7-12 mL, the volumes vary depending on the experimental conditions that the chemical robot chose.

Table S3 Summary of the starting material solutions in MeCN and their molarity.

starting material	concentration	volume	mmol	mass
**4-trifluorobenzylazide**	0.20 M	30 mL	6.0	1.208
**2-trifluorobenzylazide**	0.20 M	30 mL	6.0	1.208
**1,4-*bis*-(azidomethyl)benzene**	0.15 M	30 mL	4.5	0.847
**1,3,5-*tris*-(azidomethyl)benzene**	0.10 M	30 mL	3.0	0.730
**2-pyridinecarboxaldehyde**	0.20 M	30 mL	6.0	0.643
**4-pyridinecarboxaldehyde**	0.20 M	30 mL	6.0	0.643
**propargylamine**	0.20 M	30 mL	6.0	0.330
**3-butynylamine**	0.20 M	30 mL	6.0	0.415
**Iron (II) perchlorate hydrate**	0.10 M	30 mL	3.3	0.849
**Cobalt (II) perchlorate hydrate**	0.10 M	30 mL	3.0	1.098

4.4.1 Isolation of [Cu(2-pyridinecarboxyaldehyde)$_2$]$_n$

2-pyridinecarboxaldehyde (4.5 mL), propargylamine (1.9 mL), 4-trifluoromethylbenzylazide (2.0 mL) were mixed together. In this case the aldehyde, amine and monoazide selected were combined with the ratio 2.37:1.05:1.00 respectively. 1.6 mL of MeCN were added in order to introduce exactly 10.0 mL of this mixture to the heated copper coil (35 °C). After 40 min, the solution was collected from the copper coil and its pH, UV-Vis and ESI-MS spectra were analysed. Because of technical issues with the second step, only the ligand solution has been analyzed and left for crystallization. Blue single crystals (11 mg) were formed after six days at 18 ºC. These crystals were analyzed by X-ray diffraction and identified with the molecular structure of a copper-containing coordination polymer previously isolated by Żurowska *et al.*[10] (**Figure S16**).

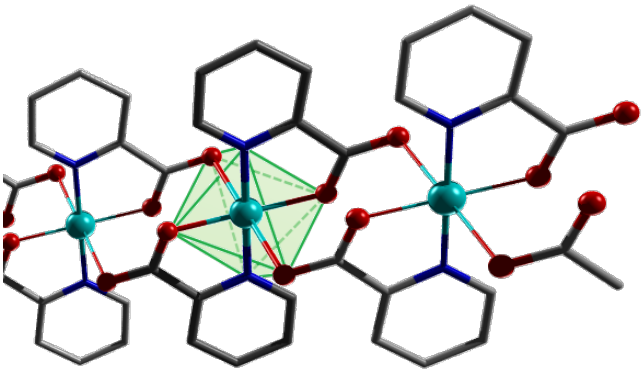

Figure S16 – Crystal structure of [Cu(2-pyridinecarboxyaldehyde)$_2$]$_n$

4.4.2 Isolation of [Fe(L^1)$_2$](ClO4)$_2$ (Complex 1)

2-pyridinecarboxaldehyde (3.2 mL), 3-butynylamine (4.7 mL), 2-trifluoromethylbenzylazide (2.5 mL) were mixed together. In this case the aldehyde, amine and monoazide selected were combined with the ratio 1.28:1.88:1.00 respectively. 10.0 mL of this mixture were pumped to the copper coil, heated at 60 °C. After 70 min, the solution was pushed out of the copper coil and its pH, UV-vis and ESI-MS spectra were collected. Iron(II) perchlorate hydrate (4.2 mL) was added and the complex solution was analyzed by pH, ESI-MS and UV-vis spectroscopy. This complex solution was collected for crystallization. After four days at 18 ºC by slow evaporation of the solvent, microcrystalline material was formed. X-ray quality crystals were grown from vapor diffusion of hexane into acetonitrile and the molecular structure identified as [Fe($\mathbf{L}^1$)$_2$](ClO_4)$_2$ (complex **1**) by X-ray diffraction analysis (14 mg, 13.73 µmol, 5.5 %). The crystallography data of complex **1** is summarized in **Section 4.1**.

[Fe(L^1)$_2$](ClO_4)$_2$ (1): ^{1}H-NMR: (CD_3CN, 400 MHz): δ = 9.58 (s, 2H, C*H*N), 8.06 (d, ^3J = 8.0 *Hz*, 2H, C*H*Ar), 7.94 (d, ^3J = 5.3 *Hz*, 2H, C*H*Ar), 7.85 (t, ^3J = 7.5 *Hz*, 2H, C*H*Ar), 7.75-7.73 (m, 2H, C*H*Ar), 7.70 (s, 2H, C*H*triazole), 7.59-7.57 (m, 4H, C*H*Ar), 7.22 (t, ^3J = 7.1 *Hz*, 2H, C*H*Ar), 7.06-7.01 (m, 2H, C*H*Ar), 5.58 (d, ^2J = 15.5 *Hz*, 2H, C*H*H), 5.53 (d, ^2J = 15.5 *Hz*, 2H, C*H*H), 4.71-4.68 (m, 2H, CH_2), 3.20-3.16 (m, 2H, CH_2) ppm. **^{13}C-NMR:** (CD_3CN, 100 MHz): δ = 172.2 (CHN), 158.8 (C_qAr), 155.3 (CHAr), 149.3 (C_qAr), 137.9 (CHAr), 133.5 (CHAr), 132.4 (C_qAr), 132.1 (CHAr), 130.0 (CHAr), 126.9 (C_qAr), 128.7 (CHAr), 127.4 (CHAr), 126.7 (CHAr), 125.2 (CHAr), 58.1 (CH_2), 52.1 (CH_2) ppm. **Benchtop ESI-MS:** *m/z* 387.25 [Fe($\mathbf{L}^1$)$_2$]$^{2+}$. **Elemental analysis:** for $C_{36}H_{32}F_6FeN_{10}Cl_2O_8.H_2O$ calcd (%) = C 43.61, H 3.45, N 14.13; found = C 43.98, H 3.35, N 13.89. **UV-vis:** (MeCN) λ_{max}, nm: 356, 476, 544. **pH of reaction mixture:** 4.34.

4.4.3 Isolation of $[Fe(L^2)_2](ClO_4)_2$ (Complex 2)

2-pyridinecarboxaldehyde (3.3 mL), propargylamine (3.3 mL), 4-trifluoromethylbenzylazide (3.3 mL) were mixed together. In this case the aldehyde, amine and monoazide selected were combined with the ratio 1.00:1.00:1.00 respectively. 0.1 mL of MeCN were added in order to introduce exactly 10.0 mL of this mixture in the copper coil heated at 80 °C. After 60 min, the solution was pushed out of copper coil and its pH, and UV-vis and ESI-MS spectra were collected. Iron(**II**) perchlorate hydrate (4.0 mL) was added and the reaction solution was again analyzed by pH, ESI-MS and UV-vis spectroscopy. The complex solution was collected for crystallization. After six days at 18 ºC single crystals were formed and the molecular structure identified as $[Fe(\mathbf{L^2})_2](ClO_4)_2$ (**2**) by X-ray diffraction analysis (13 mg, 11.66 µmol, 3.5 %). Crystallography data of complex **2** is reported in **Section 4.2**.

$[Fe(L^2)_2](ClO_4)_2$ (2): ^{1}H-NMR: (CD_3CN, 400 MHz): δ = 10.07 (t, *J* = 1.92 *Hz*, 2H, C*H*N), 8.21 (d, ^3J = 7.8 *Hz*, 2H, C*H*Ar), 7.93 (dt, ^3Jt = 7.8 *Hz*, ^4Jd = 1.4 *Hz*, 2H, C*H*Ar), 7.92 (s, 2H, C*H*triazole), 7.77 (d, ^3J = 5.5 *Hz*, 2H, C*H*Ar), 7.50 (d, ^3J = 8.2 *Hz*, 4H, C*H*Ar), 7.26 (ddd, ^3J = 7.8 *Hz*, ^3J = 5.5 *Hz*, ^4J = 1.4 *Hz*, 1H, C*H*Ar), 7.05 (d, ^3J = 8.2 *Hz*, 4H, C*H*Ar), 6.18 (d, ^2J = 20.8 *Hz*, 2H, C*H*H), 6.03 (d, ^2J = 20.8 *Hz*, 2H, C*H*H), 5.42 (s, 4H, CH_2) ppm. **^{13}C-NMR:** (CD_3CN, 100 MHz): δ = 171.3 (CHN), 160.1 (C_qAr), 154.7 (C_qAr), 150.4 (C_qAr), 139.3 (C_qAr), 138.6 (CHAr), 128.8 (CHAr), 128.7 (CHAr), 127.6 (CHAr), 126.2 (CHAr), 123.1 (CHAr), 58.8 (CH_2), 54.9 (CH_2) ppm. **Benchtop ESI-MS:** *m/z* 373.38 $[Fe(\mathbf{L^2})_2]^{2+}$. **Elemental analysis:** for $[Fe(L^2)_2](ClO_4)_2$ (= $C_{34}H_{28}F_6FeN_{10}Cl_2O_8.CH_2Cl_2$) calcd (%) = C 40.80, H 2.93, N 13.59; found = C 41.07, H 3.11, N 13.85. Solvent (CH_2Cl_2) lost and different from crystallography formula.

4.4.4 Isolation of $[Co_2(L^3)_2](ClO_4)_4$ (Complex 3)

2-pyridinecarboxaldehyde (2.3 mL), 3-butynylamine (4.4 mL), 1,4-*bis*-(azidomethyl)benzene (4.3 mL) were mixed together. In this case the aldehyde, amine and monoazide selected were combined with the ratio 1.91:1.00:1.40 respectively. 10.0 mL of this mixture were pumped to the copper coil, heated at 80 °C. After 50 min, the solution was pushed out of copper coil and its pH, UV-vis and ESI-MS spectra were collected. Cobalt(II) perchlorate hydrate (2.4 mL) was added and the reaction solution was analyzed by pH, ESI-MS and UV-vis spectroscopy. The complex solution was collected for crystallization and methanol was added. After three days at 18ºC single crystals were formed and the molecular structure identified as $[Co_2(\mathbf{L}^3)_2](ClO_4)_4$ (**3**) by X-ray diffraction analysis (2 mg, 1.08 µmol, 0.5 %). The crystallography data of complex **3** is reported in **Section 4.3**.

$[Co_2(L^3)_2](ClO_4)_4$ (3): ^{1}H NMR: (CD_3CN, 400 MHz) δ 9.71 (s, 4H, C*H*N), 8.17 (d, ^3J = 7.7 Hz, 4H, C*H*Ar), 8.04 (d, ^3J = 15.92, 4H, C*H*Ar), 7.91 (t, ^3J = 7.1 Hz, 4H, C*H*Ar), 7.70 (s, 4H, C*H*triazole), 7.32 – 7.16 (m, 4H, C*H*Ar), 7.07 (s, 8H, C*H*Ar), 5.35 (d, ^3J = 14.5 Hz, 4H, C*H*H), 5.28 (d, ^3J = 14.5 Hz, 4H, C*H*H), 4.94 – 4.30 (m, 8H, CH_2), 3.28 – 2.71 (m, 8H, CH_2). **^{13}C NMR**: (100 MHz, CD_3CN) δ 171.6 (*C*HN), 158.2 (C_qAr), 154.9 (*C*HAr), 148.4 (C_qAr), 137.6 (*C*HAr), 135.4 (C_qAr), 129.1 (*C*HAr), 128.3 (*C*HAr), 126.3 (*C*HAr), 124.2 (*C*HAr), 57.3 (CH_2), 54.1 (CH_2), 23.1 (CH_2) ppm. **ESI-MS:** *m/z* 281.27 $[Co_2(\mathbf{L}^3)_2]^{4+}$, 408.70 $\{[Co_2(\mathbf{L}^3)_2](ClO_4)\}^{3+}$, 662.12 $\{[Co_2(\mathbf{L}^3)_2](ClO_4)_2\}^{2+}$, 1423.21 $\{[Co_2(\mathbf{L}^3)_2](ClO_4)_3\}^{+}$. **Elemental analysis:** for $[Co_2(L^3)_2](ClO_4)_4$ (= $C_{56}H_{56}Cl_4Co_2N_{20}O_{16}.3.5CH_2Cl_2$) calcd (%) = C 39.22, H 3.49, N 15.37; found = C 39.16, H 3.50, N 14.76.

4.4.5 Isolation of $[Fe_2(L^3)_2](ClO_4)_4$ (Complex 4)

2-pyridinecarboxaldehyde (1.1 mL), 3-butynylamine (2.8 mL), 1,4-*bis*-(azidomethyl)benzene (2.8 mL) were mixed together. In this case the aldehyde, amine and monoazide selected were combined with the ratio 1.00:2.55:1.90 respectively. 3.3 mL of MeCN were added in order to introduce 10.0 mL of this mixture to the copper coil, heated at 70 °C. After 60 min, the solution was pushed out of copper coil and its pH, and UV-vis and ESI-MS spectra were collected. Iron(II) perchlorate hydrate (3.9 mL) was added and the reaction solution was analyzed by pH, ESI-MS and UV-vis spectroscopy. The complex solution was collected for crystallization. After six days at 18 ºC single crystals were formed and the molecular structure identified as $[Fe_2(\mathbf{L^3})_2](ClO_4)_4$ (**4**) by X-ray diffraction analysis (8.7 mg, 5.60 µmol, 5.1 %). The crystallography data of complex **4** is summarized in **Section 4.4**.

$[Fe_2(L^3)_2](ClO_4)_4$ (4): ^{1}H-NMR: (CD_3CN, 400 MHz): δ = 9.70 (s, 4H, C*H*N), 8.17 (d, ^3J = 7.7 *Hz*, 4H, C*H*Ar), 8.03 (d, ^3J = 5.6 *Hz*, 4H, C*H*Ar), 7.91 (dt, ^3Jt = 7.7 *Hz*, ^4Jd = 1.2 *Hz*, 4H, C*H*Ar), 7.68 (s, 4H, C*H*triazole), 7.30-7.27 (m, 4H, C*H*Ar), 7.07 (s, 8H, C*H*Ar), 5.34 (d, ^2J = 14.0 *Hz*, 4H, C*H*H), 5.21 (d, ^2J = 14.0 *Hz*, 4H, C*H*H), 4.75-4.69 (m, 4H, C*H*H), 4.65-4.59 (m, 4H, C*H*H), 3.15 (ddd, ^2J = 16.6 *Hz*, ^3J = 7.8 *Hz*, ^3J = 3.1 *Hz*, 4H, C*H*H), 3.00 (ddd, ^2J = 16.6 *Hz*, ^3J = 7.8 *Hz*, ^3J = 3.1 *Hz*, 4H, C*H*H) ppm. **^{13}C-NMR:** (CD_3CN, 100 MHz): δ = 172.3 (CHN), 158.8 (C_qAr), 155.6 (CHAr), 149.1 (C_qAr), 138.3 (CHAr), 136.0 (C_qAr), 129.8 (CHAr), 129.0 (CHAr), 127.0 (CHAr), 124.8 (CHAr), 58.0 (CH_2), 54.8 (CH_2), 23.8 (CH_2) ppm. **ESI-MS:** *m/z* 280.11 $[Fe_2(\mathbf{L^3})_2]^{4+}$, 407.46 $\{[Fe_2(\mathbf{L^3})_2](ClO_4)\}^{3+}$, 660.16 $\{[Fe_2(\mathbf{L^3})_2](ClO_4)_2\}^{2+}$, 1420.29 $\{[Fe_2(\mathbf{L^3})_2](ClO_4)_3\}^{+}$. **Elemental analysis:** for $[Fe_2(L^3)_2](ClO_4)_4.(H_2O)_2$ (= $C_{56}H_{56}Cl_4Fe_2N_{20}O_{16}.CH_2Cl_2$) calcd (%) = C 42.69, H 3.65, N 17.47; found = C 42.79, H 3.87, N 17.21.

4.5 OBSERVED COMPLEXES

The syntheses reported in this section did not yield any crystals. The proposed complexes are the results of ESI-MS and UV-vis analyses. In particular, evidence of the proposed complexes observed in the ESI-MS spectra is highlighted.

4.5.1 Observation of [Fe(L^{13})$_2$](ClO4)$_2$ (Complex 5)

2-pyridinecarboxaldehyde (4.9 mL), propargylamine (1.3 mL), 2-trifluoromethylbenzylazide (3.5 mL) were mixed together. In this case the aldehyde, amine and monoazide selected were combined with the ratio 3.77:1.00:2.69 respectively. 0.3 mL of MeCN were added in order to introduce exactly 10.0 mL of this mixture to the copper coil, heated at 30 °C. After 80 min, the solution was pushed out of copper coil and its pH, and UV-vis and ESI-MS spectra were collected. Iron(**II**) perchlorate hydrate (1.9 mL) was added and the reaction solution was again analyzed by pH, ESI-MS and UV-vis spectroscopy. The analysis of the solutions of these two reaction steps are summarized in **Table S4**. In particular, by ESI-MS it is possible to observe in the ligand solution the presence of a Cu complex (**5***) analogue to the assumed Fe complex (**5**).

Table S4 Summary of the analyses of the ligand and complex mixture of the reaction herein reported.

In-line analyses	Ligand solution	Complex solution
**pH**	6.05	3.69
**UV-vis**	288, 489 nm	473, 549 nm
**ESI-MS**	346.18 and 376.80 m/z<br>[**L**^{13}**+H**]$^+$ and [Cu(**L**^{13})$_2$]$^{2+}$	373. 10 m/z<br>[Fe(**L**^{13})$_2$]$^{2+}$

4.5.2 Observation of $[Co(L^1)_2](ClO_4)_2$ (Complex 6)

2-pyridinecarboxaldehyde (3.8 mL), 3-butynylamine (3.3 mL), 2-trifluoromethylbenzylazide (1.3 mL) were mixed together. In this case the aldehyde, amine and monoazide selected were combined with the ratio 2.92:2.54:1.00 respectively. 0.3 mL of MeCN was added in order to transfer exactly 10.0 mL of this mixture to the copper coil, heated at 70 °C. After 115 min, the solution was pushed out of copper coil and its pH, and UV-vis and ESI-MS spectra were collected. Cobalt(**II**) perchlorate hydrate (1.5 mL) was added and the reaction solution was analyzed by pH, ESI-MS and UV-vis spectroscopy. The analysis of the solutions of these two reaction steps are summarized in **Table S5**. In particular, by ESI-MS it is possible to observe in the ligand solution the presence of a Cu complex (**6***) analogue to the assumed Co complex (**6**).

Table S5 Summary of the analyses of the ligand and complex mixture of the reaction herein reported.

In-line analyses	Ligand solution	Complex solution
**pH**	6.74	7.73
**UV-vis**	264 nm	337 and 454 nm
**ESI-MS**	388.81 m/z $[Cu(\mathbf{L^1})_2]^{2+}$	390.85 m/z $[Co(\mathbf{L^1})_2]^{2+}$

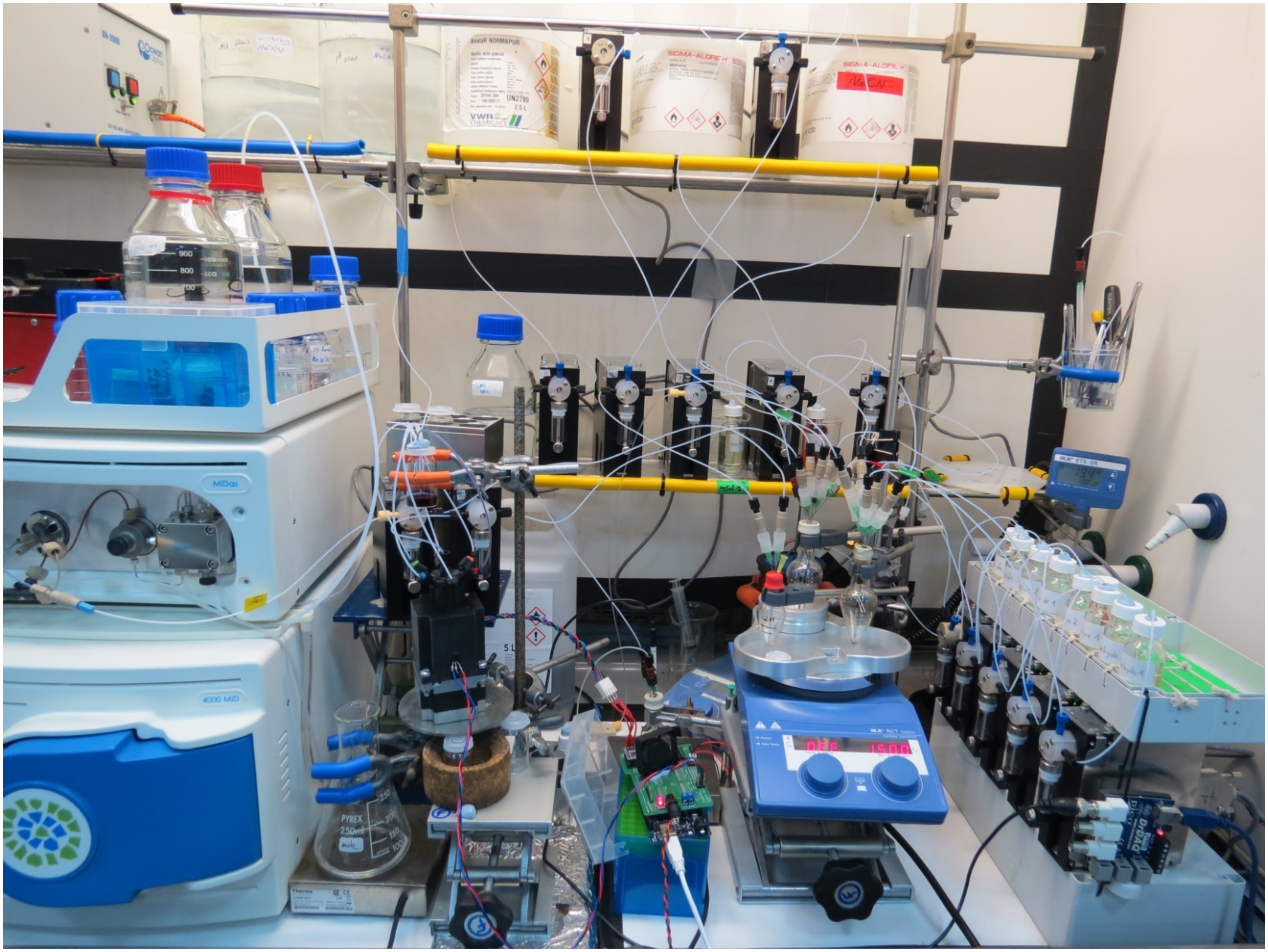

Figure S17 – Photograph of the chemical robot platform.

5 CRYSTALLOGRAPHIC DETAILS

5.1 [Fe(L[1])$_2$](ClO$_4$)$_2$·(C$_6$H$_{14}$)$_{0.5}$ (1)

Compound	**Complex 1**	
Empirical formula	$C_{36}H_{32}F_6FeN_{10}\cdot 2(ClO_4)\cdot C_3H_7$	
Formula weight	1016.55	
Temperature (K)	150(2)	
Wavelength (Å)	0.71073	
Crystal system	Monoclinic	
Space group	*P*2$_1$/*n*	
Unit cell dimensions	*a* (Å) = 16.9409(9) Å	α = 90º
	b (Å) = 15.7778(9) Å	β = 113.628(3)º
	c (Å) = 17.1724(8) Å	γ = 90º
Volume (Å^3)	4205.2(4)	
Z	4	
Calculated density (mg/m^3)	1.610	
Absorption coefficient (mm^{-1})	0.581	
F(000)	2088	
Crystal size	0.100 x 0.060 x 0.030 mm	
Theta range for data collection	1.828 to 26.000º	
Limiting indices	-20<=*h*<=18, -14<=*k*<=19, -21<=*l*<=16	
Reflections collected / unique	27449 / 7637 [R(int) = 0.0612]	
Completeness to theta = 25.242	92.6 %	
Absorption correction	Empirical	
Max. and min. transmission	1.000 and 0.857	
Refinement method	Full-matrix least-squares on F^2	
Data / restraints / parameters	7637 / 42 / 602	
Goodness-of-fit on *F*^2	1.01	
Final *R* indices [*I*>2sigma(*I*)]	*R*1 = 0.052, w*R*2 = 0.152	
R indices (all data)	*R*1 = 0.0950, w*R*2 = 0.1546	
Largest diff. peak and hole (e. Å^{-3})	0.5 and -0.49	

5.2 $[Fe(L^2)_2](ClO_4)_2$·2CH2CL2 (2)

Compound	**Complex 2**	
Empirical formula	$C_{32}H_{28}Cl_2F_6FeN_{10}O_8$.2 CH_2Cl_2	
Formula weight	1115.26	
Temperature (K)	150(2)	
Wavelength (Å)	0.71073	
Crystal system	Monoclinic	
Space group	*C*2/*c*	
Unit cell dimensions	*a* (Å) = 28.4564(17) Å	α = 90º
	b (Å) = 19.5966(11) Å	β = 106.530(3)º
	c (Å) = 18.0824(10) Å	γ = 90º
Volume ($Å^3$)	9666.9(10)	
Z	8	
Calculated density (mg/m^3)	1.533	
Absorption coefficient (mm^{-1})	0.725	
F(000)	4512	
Crystal size	0.100 x 0.050 x 0.030 mm	
Theta range for data collection	1.279 to 25.000º	
Limiting indices	-33<=*h*<=33, -23<=*k*<=23, -21<=*l*<=21	
Reflections collected / unique	125284 / 8512 [R(int) = 0.1025]	
Completeness to theta = 25.000	100.0 %	
Absorption correction	Empirical	
Max. and min. transmission	0.985 and 0.861	
Refinement method	Full-matrix least-squares on F^2	
Data / restraints / parameters	8512 / 8 / 616	
Goodness-of-fit on *F*^2	1.062	
Final *R* indices [*I*>2sigma(*I*)]	*R*1 = 0.0762, w*R*2 = 0.2190	
R indices (all data)	*R*1 = 0.1174, w*R*2 = 0.2628	
Largest diff. peak and hole (e. $Å^{-3}$)	1.68 and -0.43	

5.3 $[Co_2(L^3)_2](ClO_4)_4\cdot 10CH_3OH$ (3)

Compound	**Complex 3**	
Empirical formula	$C_{56}H_{56}Cl_4Co_2N_{20}O_{16}$.10 CH_3OH	
Formula weight	1845.28	
Temperature (K)	150(2)	
Wavelength (Å)	0.71073	
Crystal system	Triclinic	
Space group	*P*-1	
Unit cell dimensions	*a* (Å) = 12.3175(8) Å	α = 105.504(3)º
	b (Å) = 18.6326(11) Å	β = 103.148(3)º
	c (Å) = 19.8659(12) Å	γ = 98.558(3)º
Volume (Å^3)	4170.2(5)	
Z	2	
Calculated density (mg/m^3)	1.470	
Absorption coefficient (mm^{-1})	0.613	
F(000)	1924	
Crystal size	0.600 x 0.500 x 0.090 mm	
Theta range for data collection	1.108 to 25.999º	
Limiting indices	-15<=*h*<=14, -22<=*k*<=22, -24<=*l*<=24	
Reflections collected / unique	124036 / 16391 [R(int) = 0.0448]	
Completeness to theta = 25.242	100.0 %	
Absorption correction	Empirical	
Max. and min. transmission	0.871 and 0.762	
Refinement method	Full-matrix least-squares on F^2	
Data / restraints / parameters	16391 / 1359 / 901	
Goodness-of-fit on *F*^2	1.024	
Final *R* indices [*I*>2sigma(*I*)]	*R*1 = 0.0546, w*R*2 = 0.1523	
R indices (all data)	*R*1 = 0.0712, w*R*2 = 0.1714	
Largest diff. peak and hole (e. Å^{-3})	1.54 and -0.82	

5.4 $[Fe_2(L^3)_2](ClO_4)_4{\cdot}(CH_2Cl_2)_2$ (4)

**Compound**	**Complex 4**
Empirical formula	$C_{56}H_{56}Cl_4Fe_2N_{20}O_{16}.(CH_2Cl_2)_{0.8}.(CH_2Cl_2)_{1.2}$
Formula weight	1688.56
Temperature (K)	150(2)
Wavelength (Å)	0.71073
Crystal system	Monoclinic
Space group	$P2_1/n$
Unit cell dimensions	*a* (Å) = 13.8956(17) Å α = 90º<br>*b* (Å) = 25.068(3) Å β = 90.328(9)º<br>*c* (Å) = 22.547(3) Å γ = 90º
Volume (Å^3)	7853.7(16)
Z	4
Calculated density (mg/m^3)	1.428
Absorption coefficient (mm^{-1})	0.714
F(000)	3456
Crystal size	0.100 x 0.080 x 0.050 mm
Theta range for data collection	1.215 to 24.467 º
Limiting indices	-15<=*h*<=16, -29<=*k*<=26, -26<=*l*<=23
Reflections collected / unique	36656 / 12968 [*R*(int) = 0.1343]
Completeness to theta = 24.467	99.6 %
Absorption correction	Empirical
Max. and min. transmission	1.000 and 0.734
Refinement method	Full-matrix least-squares on F^2
Data / restraints / parameters	12968 / 1130 / 859
Goodness-of-fit on *F*^2	1.074
Final *R* indices [*I*>2sigma(*I*)]	*R*1 = 0.1077, w*R*2 = 0.2921
R indices (all data)	*R*1 = 0.2441, w*R*2 = 0.4077
Largest diff. peak and hole (e. Å^{-3})	1.01 and -0.50

6 REFERENCES

[1] G. Sheldrick, *Acta. Crystallogr. A* **1990**, *46*, 467-473

[2] G. Sheldrick, *Acta. Crystallogr. A* **2008**, *64*, 112-122

[3] L. Farrugia, *J. Appl. Crystallogr.* **1999**, *32*, 837-838

[4] O. V. Dolomanov, L. J. Bourhis, R. J. Gildea, J. A. K. Howard and H. Puschmann. J. Appl. Cryst. 2009, 42, 339341.

[5] R. C. Clark, J. S. Reid*, Acta. Crystallogr. A* **1995**, *51*, 887-897

[6] A.L.Spek, Acta Cryst. **2009**, D65, 148-155.

[7] S. G. Alvarez, M. T. Alvarez, *Synthesis* **1997**, *4*, 413

[8] S. Dadashi-Silab, B. Kiskan, M. Antonietti, Y. Yagci, *RSC Adv*. **2014**, *4*, 52170

[9] F. Sander, U. Fluch, J. P. Hermes, M. Mayor, *Small* **2014**, *10*, 349

[10] B. Żurowska, J. Mroziński, K. Ślepokura, *Polyhedron* **2007**, *26*, 3379
